# Magnetic Ionic Liquids: Current Achievements and Future Perspectives with a Focus on Computational Approaches

**DOI:** 10.1021/acs.chemrev.3c00678

**Published:** 2024-03-11

**Authors:** Nádia M. Figueiredo, Iuliia V. Voroshylova, Elisabete S. C. Ferreira, Jorge M. C. Marques, M. Natália D. S. Cordeiro

**Affiliations:** † LAQV@REQUIMTE, Department of Chemistry and Biochemistry, Faculty of Sciences, University of Porto, Rua do Campo Alegre, 4169-007 Porto, Portugal; ‡ CQC−IMS, Department of Chemistry, University of Coimbra, 3004-535 Coimbra, Portugal

## Abstract

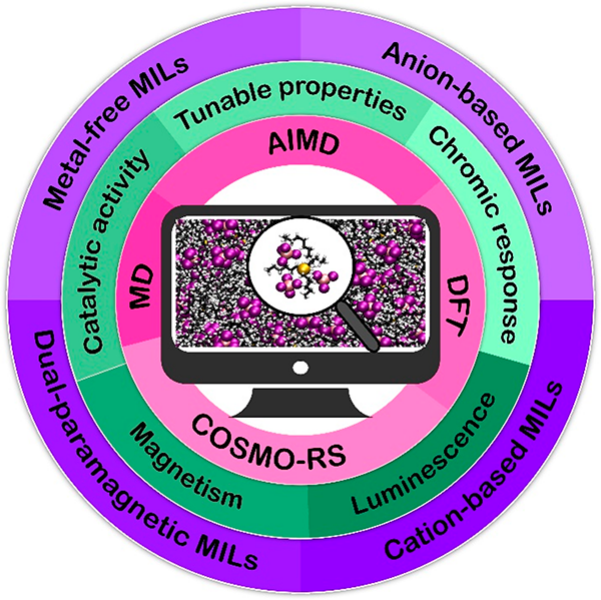

Magnetic ionic liquids (MILs) stand out as a remarkable subclass of ionic liquids (ILs), combining the desirable features of traditional ILs with the unique ability to respond to external magnetic fields. The incorporation of paramagnetic species into their structures endows them with additional attractive features, including thermochromic behavior and luminescence. These exceptional properties position MILs as highly promising materials for diverse applications, such as gas capture, DNA extractions, and sensing technologies. The present Review synthesizes key experimental findings, offering insights into the structural, thermal, magnetic, and optical properties across various MIL families. Special emphasis is placed on unraveling the influence of different paramagnetic species on MILs’ behavior and functionality. Additionally, the Review highlights recent advancements in computational approaches applied to MIL research. By leveraging molecular dynamics (MD) simulations and density functional theory (DFT) calculations, these computational techniques have provided invaluable insights into the underlying mechanisms governing MILs’ behavior, facilitating accurate property predictions. In conclusion, this Review provides a comprehensive overview of the current state of research on MILs, showcasing their special properties and potential applications while highlighting the indispensable role of computational methods in unraveling the complexities of these intriguing materials. The Review concludes with a forward-looking perspective on the future directions of research in the field of magnetic ionic liquids.

## 1 Introduction

Emerging as alternative solvents, ionic liquids (ILs) constitute a class of molten salts with melting points below 373.15 K. The widespread attention paid to ILs is mainly due to their unique and tunable physicochemical properties, such as negligible vapor pressure, nonflammability, low melting points, high stability over a wide temperature range, good conductivity, large potential window, and ability to dissolve a variety of compounds. Typically, ionic liquids consist of an organic cation with a symmetrical or asymmetrical backbone comprising one or more alkyl chains, along with an inorganic anion that can range from simple halides like Cl^–^ or Br^–^ to more complex structures. A new generation of ILs, known as task-specific ILs (TSILs), has recently arisen as a result of the growing research on ILs. In fact, the particular features of TSILs can be tailored by functionalizing one or both of the ionic species.^1^ For example, by incorporating a paramagnetic atom (commonly a *d*- or *f*-transition metal) in the anion and/or cation leads to the so-called magnetic ionic liquids (MILs) that exhibit susceptibility to external magnetic fields.^2−5^ MILs retain all of the remarkable characteristics of ILs, with the added benefit of inherent magnetic mobility. Their magnetoactive nature allows them to be easily separated and recovered through magnetic separation, which is a simple, cost-effective, and environmentally friendly way to recycle these liquids.^6−8^ This aspect is particularly advantageous as it enables their reuse without generating waste or compromising their efficiency.^9^

In 2004, the discovery of the strong magnetic properties of the 1-butyl-3-methylimidazolium tetrachloroferrate(III) ([C_4_C_1_im][FeCl_4_]) IL and of its potential applications clearly marked the onset of a new area of research interest focused on MILs.^9,10^ Fe-based MILs have been extensively studied since then, but their slight instability in aqueous media, owing to the hydrolysis of FeCl_4_^–^ anion,^11^ has raised concerns about potential side effects and loss of selectivity toward the analytes. Yet, other magnetic anions are being explored in aqueous media, and Fe-based MILs can still be used in nonaqueous media.^12−17^ To provide both hydrolytic stability and specific magnetic responses to target analytes, paramagnetic atomic centers like Mn, Ni, Co, Dy, Nd, and Gd^9,11^ are often embedded into MILs through halides, thiocyanates or perfluorinated ligands.^18,19^ Most often, MILs contain transition metal or lanthanide complexes in their anion structure. As for cations, imidazolium, phosphonium, ammonium, and pyridinium are among the most explored in MIL’s formation.^9^ N-Substituted imidazole ligands are one of the few types of MILs that use a paramagnetic core in the cation pair, and even fewer incorporate paramagnetic cores in both cation and anion pairs.^5^ Strategies for innovating MIL’s formulations have led to recent reports on a novel type of magnetic solvent in which the paramagnetic compound is an organic radical rather than a metal core, known as organic MILs.^9,20^ Indeed, there is a multiplicity of possible combinations between anions and cations that can be used to obtain MILs, with the choice depending on the physicochemical properties required for a particular application. In addition to anion/cation combinations, molecular solvents can be added to optimize the MILs’ properties, providing enhanced transport properties—i.e., lower viscosity and higher conductivity, greater hydrophobicity, and even improving their magnetic responses.^21^ Owing to this tunability, a wide range of versatile applications have already been exploited by employing MILs as custom-made designer solvents. The type of doped metal center defines their optical, thermal, and volumetric properties.^18^ For instance, MILs containing *f*-transition metals are known to also exhibit luminescence and stimuli-responsive features.^22^ The spin state (high or low spin) of the metal center also plays a role in establishing their magnetic, optical, luminescence, and chromic properties.^12^ Specifically, high-spin transition metals lead to magnetic susceptibility in MILs.^11^

MILs are typically synthesized using multistep but easy procedures. For example, anion-based MILs are commonly synthesized at room temperature without reflux,^9^ although purification methods such as solvent evaporation/recovery, recrystallization, and washing steps are required.^16,21,23^ Yet, magnetic separation can be used to remove MILs from the reaction media in a simple way.^9^ However, some metal-containing precursors, such as rare earth metals, can make such synthetic procedures more expensive than those of conventional ILs. As an alternative to MILs based on lanthanides, iron-based MILs are obviously less expensive due to the low cost and abundance of iron.^23^

Most of the applications of magnetic ionic liquids have been focused so far on extraction and separation processes.^24−35^ Several extraction and/or separation techniques can be used to obtain a compound, some of which involve large volumes of toxic organic solvents.^9,24^ Thus, increasing attention has been paid in recent years to the development of microtechniques resourcing to the use of greener solvents.^9,24^ Moreover, to cope with poor phase separation, particularly in solvent-based extractions, the use of MILs is an asset. Indeed, MILs provide a more sustainable and efficient approach to extraction and separation processes, thanks to their unique properties of being retrievable and reusable.^9,24,36^ The latter not only improves efficiency but also reduces the time and energy required for these procedures. Moreover, numerous studies have consistently shown that separation and isolation processes utilizing MILs provide several advantages compared to traditional procedures.^37^ These processes are known to be simpler and more cost-effective, while also achieving high extraction efficiency.^37^ In fact, MILs find application in the separation and extraction of various systems, ranging from metals to carbon dioxide, including organic and inorganic compounds, as well as natural compounds.^2,24,38−42^ The advantages of easy recovery provided by their magnetic responsiveness, along with their thermal and chemical stability, electrochromic and luminescent behavior, also allow for their use in catalysis, electrochemical, and sensor applications.^43−52^

Despite all the experimental research work devoted to the use of MILs, their successful industrial implementation requires a thorough understanding of their properties and behavior. Due to the multiplicity of possible combinations between the anion and cation components, careful tailoring of the MIL’s components is imperative for their successful application.

Theoretical methodologies play a crucial role in the design of MILs, offering a spectrum of approaches to investigate their fundamental properties beyond empirical data. Central to these techniques is the application of predictive molecular thermodynamics models, such as the Conductor-like Screening Model for Real Solvents (COSMO-RS), alongside a variety of empirical equations.^53−55^ These models are instrumental in elucidating the interactions, solubility, and phase behavior of MILs, for their effective design and application.^56,57^

Additionally, computational modeling is a powerful tool for elucidating the relationships that govern the suitability of MILs for specific applications, which is crucial for designing new and more effective methodologies.^55,58−60^ Actually, time and resources can be saved by understanding their electronic properties, predicting their physicochemical properties, or designing well-fitted ILs based on their structural features in bulk or mixtures with other organic solvents.^61^ A variety of scales can be explored to study these compounds at an atomistic level, ranging from quantum mechanical (QM) calculations to molecular dynamics (MD) simulations. However, achieving feasibility and accuracy in results remains a primary goal for theoretical chemists studying these compounds. Due to the complex nature of MILs and the influence of short- and long-range electrostatic forces, obtaining accurate results in atomistic-level modeling can still be challenging. Nevertheless, the growing demand for computational modeling and theoretical studies of MILs in recent decades suggests that it is a promising avenue for advancing our understanding of these liquids.

This Review will begin by introducing diverse types of MILs, exploring their distinctive structures and properties. From an experimental standpoint, the practical applications of MILs will be presented. Additionally, the Review will encompass recent theoretical and computational approaches used to unravel the structure, properties, and interactions of MILs, exploiting their potential applications in complementing experimental studies and facilitating design efforts. Finally, future perspectives on MIL studies, with a particular emphasis on computational approaches, will be provided to guide further advancements in this field.

## 2 Magnetic Ionic Liquids Structure and Properties: Insights from Experimental Studies

As pointed out, magnetic ionic liquids contain paramagnetic species, typically a transition metal or lanthanide atom, in their cation or anion structure, or both. These paramagnetic atoms have unpaired electrons in *d*- (e.g., Fe(III), Mn(II), Cu(II), etc.) and *f*-subshells (e.g., Dy(III), Gd(III), Ho(III), etc.), resulting in high magnetic moments and positive magnetic susceptibilities. As a result, MILs preserve the intrinsic characteristics of ILs while exhibiting magnetic susceptibility in the presence of an external magnetic field. MILs can incorporate a range of paramagnetic species, enabling the simultaneous manifestation of both magnetic and luminescent properties. Likewise, several MILs show stimulus-responsive attributes—i.e., chromic properties, wherein their color is modified by external stimuli like temperature, vapor, light, potentials, or solvents. These properties are advantageous as they facilitate physical separation through an external magnetic field or visual differentiation based on distinct characteristics such as color. A wide range of applications can benefit from these unique features, including separation processes in miscible media, sensing, and the development of smart materials.

However, similarly to ILs, the chemical structure of MILs plays a crucial role in determining their thermal, physicochemical, and transport properties. Therefore, it is essential to understand and rationalize their structure–property relationships in order to optimize their applicability, and performance, and to design new MILs.

The properties of MILs are influenced by various factors, including the nature, size, and asymmetry of their cation and anion components, as well as the charge delocalization of the anion. Interionic interactions, such as electrostatic effects, π-stacking, and hydrogen bonding (H-bonding), also contribute to changes in the macroscopic properties of these salts. Besides, the presence of paramagnetic atoms (transition metals or rare earth elements) incorporated in the MIL’s structure, whether in the cation, anion, or both, greatly affects their properties.

In the following subsections, we will outline the noteworthy properties and key considerations pertaining to MILs based on their structures, as derived from experimental findings.

### 2.1 Anion-Based MILs

Anion-based magnetic ionic liquids that incorporate paramagnetic atoms into the anion structure have garnered significant interest. Metals can be incorporated in the form of halogenated anions or in larger and more complex structures like acetylacetonate anions. These anions are frequently coupled with cations commonly found in ILs, such as imidazolium, phosphonium, or ammonium cations.

#### 2.1.1 Halometallates

The discovery of the magnetic response exhibited by [C_4_C_1_im][FeCl_4_] MIL opened new avenues for studying this specific class of compounds.^10,62^ Since then, the [FeCl_4_]^−^ anion has been the most studied among anion-based MILs.^12,17,46,49,63−65^ One of the primary reasons for significant attention paid to the paramagnetic properties of high-spin Fe(III) is its relatively low cost and abundance.^66^

**Figure 1:**
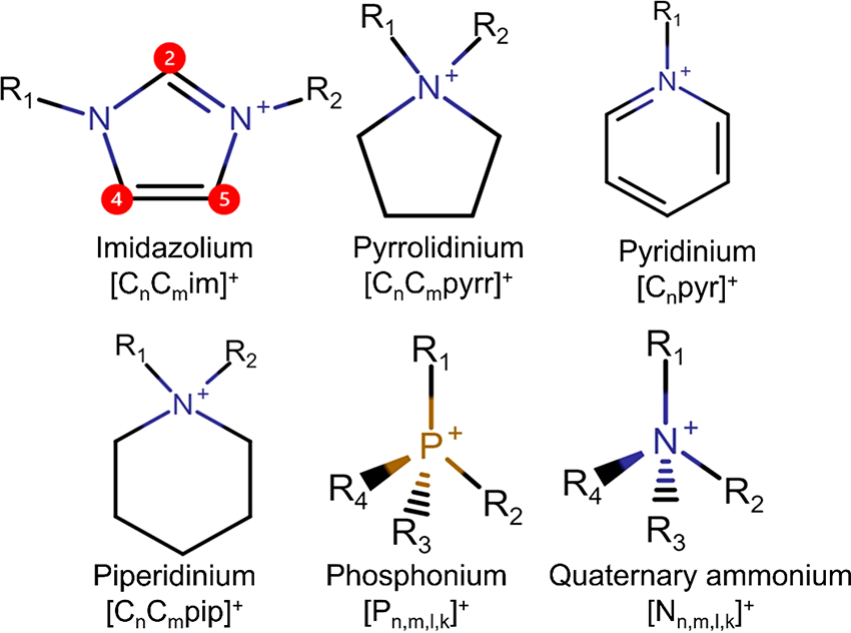
Illustration showcasing prevalent cationic frameworks found in ILs, including imidazolium [C_n_C_m_im]^+^, pyrrolidinium [C_n_C_m_pyrr]^+^, pyridinium [C_n_pyr]^+^, piperidinium [C_n_C_m_pip]^+^, phosphonium [P_n,m,l,k_]^+^, and ammonium [N_n,m,l,k_]^+^ cations. The imidazolium cation features a red-numbered system, pinpointing key sites to facilitate a deeper understanding of its structural characteristics.

Studies have demonstrated that the MIL 1-ethyl-3-methylimidazolium tetrachloroferrate(III), [C_2_C_1_im][FeCl_4_], exhibits several noteworthy properties. It has a melting point below room temperature, shows a magnetic response in the presence of a small neodymium magnet, and displays a low viscosity (14 mPa s^–1^ at 293 K) as well as a good conductivity (1.8 × 10^–2^ S cm^–1^ at 303 K).^67^ Its imidazolium cation is generally considered unpopular due to the presence of the C2–H bond (see Figure 1), which is the most acidic site in the imidazolium ring and prone to forming H-bonds with the anions. However, the analysis of the crystal structure of [C_2_C_1_im][FeCl_4_] revealed that the C2–H bond does not interact with the [FeCl_4_]^−^ anion.^68^ Based on the infrared spectrum analysis, it appears that the key bands are associated with the C4–H and C5–H bonds, possibly due to the acidic Lewis character of the [FeCl_4_]^−^ anion.^15^

The elongation of the imidazolium cation and the replacement of the halide atom in the anion structure have also been investigated.^15^ When alkyl chains are added to imidazolium-based MILs with [FeCl_4_]^−^ and [FeBr_4_]^−^ anions, their viscosities increase while their conductivities decrease. This effect is primarily attributed to the van der Waals attraction between the imidazolium chains, whereas replacing chloride with bromide in the anion structure results in an increase in both the melting point and viscosity of the MIL. This behavior is attributed to the bromine atoms in the anion structure having more significant cloud expanding.^15^ Interestingly, similar magnetic susceptibilities (i.e., ranging from 1.38 to 1.44 × 10^–2^ emu mol^–1^) are observed for [C_*n*_C_1_im][FeCl_4_] and [C_*n*_C_1_im][FeBr_4_], where *n* represents the length of the alkyl chain (*n* = 2, 4, 6, and 8). It is worth noting here that shorter alkyl chains, such as in [C_2_C_1_im]^+^, enhance the efficiency of magnetic interaction.^15^ Additionally, the presence of antiferromagnetic order below de Néel temperature of *ca*. 3.8 K has also been confirmed.^12^

Even if these halometallate MILs have desirable transport properties like low viscosity and good conductivity, they are quite sensitive to water and oxygen, which limits their versatility. For instance, when using MILs in microextraction procedures, it is necessary to skeletonize their water solubility.^3^ Nonetheless, they can still be useful for applications that do not involve aqueous media^39,69^ and can be recovered using a magnetic field.^70^

Hydrophobic MILs containing halometallate anions have been specially tailored to improve their performance.^13,21,71−73^ For example, the hydrophobic phosphonium-based MIL, trihexyl(tetradecyl) phosphonium tetrachloroferrate(III) ([P_66614_][FeCl_4_]), exhibits more desirable properties compared to other MILs from the imidazolium family. [P_66614_][FeCl_4_] has a lower density of 0.989 g cm^–3^ at 298.15 K, positive magnetic susceptibility, and higher viscosity of 1349 mPa s^–1^ at 298.15 K. The elongated alkyl chains in the phosphonium cations contribute to the higher viscosity compared to the imidazolium-based MILs. Generally, longer alkyl chains lead to lower densities and higher viscosities in the MILs. However, the viscosity of [P_66614_][FeCl_4_] decreases significantly with increasing temperature. For example, at 373.15 K, the viscosity drops to 40.7 mPa s^–1^.^10^ Furthermore, Santos et al.^74^ have found that the viscosity of [P_66614_][FeCl_4_] decreases with increasing external magnetic field strength. In this case, the viscosity falls from 749 mPa s^–1^ in the absence of the magnetic field to 672 mPa s^–1^ in the presence of a 2 T magnetic field.^74^ This behavior has also been observed in other halometallate-based MILs.^74−77^ However, to the best of our knowledge, an explanation for the transport properties change in the presence of a magnetic field has not yet been established.

The magnetic responsivity of MILs not only facilitates magnetic extraction but also has a positive impact on their transport properties. In a study involving imidazolium-family cations and [FeCl_4_]^−^ anions, an external magnetic field was applied to investigate its effects on the transport of organic compounds through supported magnetic ionic liquid membranes.^75^ The results showed that the diffusion coefficients of 1-butyl-3-methylimidazolium tetrachloroferrate(III) ([C_4_C_1_im][FeCl_4_]) and of 1-octyl-3-methylimidazolium tetrachloroferrate(III) ([C_8_C_1_im][FeCl_4_]) increased when a magnetic field was applied. Additionally, the viscosity of the MILs decreased with increasing magnetic field strength. Specifically, the viscosity of [C_4_C_1_im][FeCl_4_] decreased by 6.6% for 1.2 T and 10.3% for 2.0 T, while the viscosity of [C_8_C_1_im][FeCl_4_] decreased by 8.1% for 1.2 T and 20.1% for 2.0 T, in response to varying magnetic field intensities measured in Tesla (T).^75^ These findings support previous observations of improved performance in the presence of a magnetic field, including when [FeCl_4_]^−^ is paired with alkylphosphonium cations.^77^

Several cations have been combined with the [FeCl_4_]^−^ anion to form MILs, including those from dialkylimidazolium-, alkylamommonium-, pyridinium-, and pyrrolidinium-based families.^17,50,78,79^ These MILs generally exhibit good thermal stability but are not liquid at room temperature. Additionally, other chlorometallate anions, such as tetrachloromanganate(II) ([MnCl_4_]^2–^), tetrachlorocobaltate(II) ([CoCl_4_]^2–^) and hexachlorogadolinium(III) ([GdCl_6_]^3–^) when combined with the [P_66614_] cation result in extremely high viscosities under ambient conditions (75230, 83450, and 18390 mPa s^–1^, respectively).^71^ For example, [P_66614_]_3_[GdCl_6_] exhibits resistance to hydrolysis in aqueous media, low UV background, and rapid recovery of the extraction media when a strong magnet is used.^73^ Furthermore, lanthanide atoms show a significant improvement in magnetic susceptibility compared to transition metal components in halometallate anions.^80^

The incorporation of rare earth elements into halometallate anions has been found to enhance both the luminescence and the magnetic susceptibility.^4,81−83^ MILs such as 1-dodecyl-3-methylimidazolium hexabromodysprosiate(III) [C_12_C_1_im]_3_[DyBr_6_] exhibit high luminescence, thanks to the *f*–*f* transition characteristics of the trivalent Dy atom (4*f*^9^ electron configuration), as well as to the imidazolium cation that acts as a sensitizer to activate the lanthanide atom. Under excitation by a conventional UV lamp, either a bright white or orange–yellow emission can be observed. Additionally, magnetic measurements at 298.15 K indicate a magnetic moment (μ_eff_) of 9.6 μ_B_, allowing for easy manipulation of the MILs with an external magnetic field.^82^

Del Sesto and co-workers synthesized hexachlorolanthanide(III)-based MILs, [P_66614_]_3_[LnCl_6_], where Ln represents Tb(III), Dy(III), Ho(III), and Er(III). These MILs exhibited lower viscosities ranging from 2000 to 2500 mPa s^–1^ at 300 K. The magnetic susceptibility of [LnCl_6_] anions varied from 14.3 to 11.2 emu K mol^–1^, with [HoCl_6_]^3–^ > [DyCl_6_]^3–^ > [TbCl_6_]^3–^ > [ErCl_6_]^3–^. Interestingly, the magnetic behavior remained unchanged at the glass transition temperature (200 K), suggesting the presence of intermediate structures during glass formation that influence the magnetic properties. As such, the presence of phosphonium cations contributed to the glassy behavior of the MILs without crystallization, while the incorporation of rare earth metals enhanced their magnetic properties.^84^

Recently another halometallate anion, the bromotrichloroferrate heteroanion, [FeCl_3_Br]^−^, has been studied and applied in a series of MILs.^66,85−87^ By pairing [FeCl_3_Br]^−^ with symmetrical and unsymmetrical dicationic and tricationic quaternary ammonium cations, MILs with remarkable properties were obtained. In their study, Nacham et al.^23^ focused on quaternary ammonium cations lacking acid protons and synthesized three types of hydrophobic MILs. Introducing symmetry in the quaternary ammonium-based MILs (i.e., benzylimidazolium substituents) led to lower melting points. Even after replacing the asymmetric hexadecylbenzimidazolium substituent with benzylimidazolium, the melting point was further reduced without compromising hydrophobicity or magnetic susceptibility. This can be explained by the removal of symmetry in the cationic part and the scarcity of π–π interactions. However, tricationic MILs comprising [FeCl_3_Br]^−^ anions exhibited high effective magnetic moments (μ_eff_) of 11.76 Bohr magnetons (μ_B_), comparable to the values only previously achieved with lanthanide-based MILs. Previously, such high μ_eff_ values could only be achieved using lanthanides in MILs structure. This indicates that Fe-containing MILs can serve as a cost-effective alternative to lanthanide-based systems.^23^

The properties of [FeCl_3_Br]^−^ anion combined with symmetric ([(C_*n*_)_2_im]^+^, *n* = ethyl, butyl, hexyl, octyl, decyl, dodecyl) and asymmetric ([C_*n*_C_1_im], *n* = ethyl, butyl, hexyl, octyl, decyl, dodecyl) imidazolium cations were also evaluated.^66^ In the air, these compounds exhibited short-term thermal stability above 573 K in ambient conditions. Surprisingly, the melting points of these MILs were low (below room temperature) despite the presence of symmetrical cations, which typically contribute to higher melting points in ILs.^66,88^ X-ray crystal structure analysis suggested that bulky anions may disrupt crystalline order, resulting in lower melting points. The densities of [(C_2_)_2_im][FeCl_3_Br] and [C_2_C_1_im][FeCl_3_Br] were found to be 1.573 and 1.627 g cm^–3^, respectively, and the kinematic viscosities range from 12.8 to 42.6 cSt depending on the alkyl chain length in the cations. MILs based on [(C_*n*_)_2_im]^+^ cations exhibited greater viscosity increases with elongation of the alkyl chain, attributed to the growing molecular size of the cation.^66^

In summary, the choice of weakly coordinating anions and the incorporation of rare earth metals instead of transition metals can significantly lower the viscosity of phosphonium-based MILs. Also, halometallate-based MILs with different paramagnetic atoms offer distinct characteristics such as magnetic susceptibility, Lewis acidity, thermal stability, and viscosity.

#### 2.1.2 Fluorinated Acetylacetonates

Recently, Pierson et al.^21^ synthesized a series of hydrophobic MILs with hexafluoroacetylacetonate (hfac) chelated metal anions paired with the [P_66614_]^+^ cation, namely, [P_66614_][M(hfac)_*x*_], where M represents Co(II), Mn(II), Ni(II), Dy(III), Gd(III) and Nd(III). These MILs exhibited relatively low viscosities (276.5–927.9 mPa s^–1^) compared to those containing tetrachlorometalate anions, making them easier to handle. Interestingly, MILs containing transition metals showed higher viscosities than those containing lanthanides. Both the decrease in atomic radii and metal–ligand distances contribute to a reduction in intermolecular strength and an increase in viscosity. Additionally, the presence of bulkier anions in rare earth-based MILs reduces packing and intermolecular forces, lowering their viscosity. As expected, MILs based on Dy(III) and Gd(III) anions exhibited high magnetic susceptibility, a characteristic inherent to rare earth metals.^21^

Lu et al.^89^ utilized 1-decyl-3-methylimidazolium hexafluoroacetylacetonate chelated metal anions ([C_10_C_1_im][M(hfac)_3_], where M = Co(II), Ni(II), and Cu(II)) to incorporate divalent metals into the anions. The melting points of the resulting MILs ranged from 311 to 318 K, and their densities at 328.15 K varied from 1.374 to 1.395 g cm^–3^. MILs containing copper exhibited the highest density, followed by those containing cobalt and nickel. However, the density did not significantly change with variations in the chelated metal anion structure. Similar trends were observed for other volumetric properties such as molecular volume, coefficient of thermal expansion, and molar entropy. According to the authors, the large volume of the anion causes a reduction in the electric charge density and weakens the interaction in the ion pair, leading to these observations. The long alkyl chain of the imidazolium cation also makes its arrangement difficult. The viscosity of these compounds, however, differed significantly, with, *e.g*., [C_10_C_1_im][Ni(hfac)_3_] exhibiting significantly higher viscosity (192.52 mPa s^–1^) than [C_10_C_1_im][Cu(hfac)_3_] (109.95 mPa s^–1^) at 328 K. Lower electrical conductivity and surface tension were also observed at temperatures between 323.15 and 343.15 K. Likewise, these compounds showed lower fusion enthalpy (21.71–26.24 kJ mol^–1^) and fusion entropy (69.72–82.57 J mol^–1^ K^–1^).^89^

In another study, the physicochemical, transport, and magnetic properties of a series of 24 MILs have been examined by varying their divalent metal centers (Ni(II), Co(II), and Mn(II)), aromatic portions, and ion-pair combinations.^90^ In such MILs, long alkyl chain cations such as [P_66614_]^+^ and 1-tetradecyl-3-methylimidazolium ([C_14_C_1_im]^+^) were used. It has been observed that incorporating aromatic portions into the acetylacetonate anion significantly improved the thermal stability of the MILs. The combination of cations and anions resulted also in diverse viscosity values, ranging from 100 mPa s^–1^ to *ca*. 50000 mPa s^–1^ at 338 K. The addition of 1,1,1-trifluoro-2,4-pentanedione was found to reduce viscosities for MILs with both cations. Remarkably, MILs with phosphonium-derived cations exhibited lower viscosities (119 to 172 mPa s^–1^ at 338 K) than the ones with imidazolium-based cations (845 to 2209 mPa s^–1^). In contrast, phosphonium and imidazolium cations paired with halometallates behaved differently. However, acetylacetonate-based MILs were found to be soluble in many organic solvents, making them versatile and suitable for high-temperature applications.^90^

One more study focused on chelating Co(II), Cu(II), Mn(II), and Ni(II) atoms into the [M(hfac)] anion and forming corresponding MILs with tetrabutylammonium ([N_4444_]^+^), *n*-tetradecylpyridinium ([C_14_pyr]^+^) and [C_10_mim]^+^ cations. The MILs with an imidazolium cation showed characteristics similar to typical room-temperature ionic liquids (ILs) and had higher densities (ranging from 1.34 to 1.44 g cm^–3^). The melting points of MILs with cations from the ammonium family ranged from 339 to 346 K, whereas the introduction of pyridinium-family representatives resulted in melting around 310 K. The highest melting point was observed for the Co(II) chelated anion, while the lowest was achieved for the Mn(II)-based MIL. The presence of transition metals was found to increase the thermal stability of the MILs compared to the metal-free [C_10_C_1_im][hfac] IL. The study also reported the possibility of extracting and reusing these MILs, as they could be recovered from mixtures and recycled up to five times without losing their catalytic usefulness.^91^

Despite the high viscosity associated with these highly coordinated anions, the presence of paramagnetic atoms within the hexafluoroacetylacetonate moiety enhanced the hydrophobicity and air stability of the corresponding MILs. These characteristics make them well-suited for high-temperature requirements.

#### 2.1.3 Iso- and Thiocyanatometallates

Thiocyanate and isothiocyanate anions are some of the most common anions in conventional ILs. In the case of MILs, thiocyanate anions have different stoichiometries when a paramagnetic atom is present, for example, the MIL [C_4_C_1_im]_2_[Co(SCN)_4_] (2:1), in which two imidazolium cations are coupled with the cobalt-thiocyanate anion.^92^ Del Sesto et al. were the first to our knowledge to report the insertion of a paramagnetic atom into the thiocyanate anion structure.^93^ In their study, bulky phosphonium cations were used to synthesize two MILs: [P_66614_]_2_[Co(SCN)_4_] and [P_66614_]_2_[Ni(SCN)_6_]. These compounds exhibited low density, measuring less than 1 g cm^–3^, and high viscosities. At a temperature of 293.15 K, the [Co(SCN)_4_]^2–^ MIL had a high viscosity of 2436 mPa s^–1^, while the [Ni(SCN)_6_]^2–^ MIL had a slightly lower viscosity of 760 m Pa s^–1^.^93^

Recently, the investigation of MILs with the [Co(SCN)_4_]^2–^ anion has been extended to include imidazolium-family cations.^18,94,95^ In particular, the MIL [C_4_C_1_im]_2_[Co(SCN)_4_] was found to exhibit a magnetic moment μ_eff_ = 4.40 μB, consistent with Co(II) systems with a spin value of 3/2. As well, the Curie–Weiss temperature, a measure of the strength of magnetic interactions, that was found (= −0.9 K) indicated very weak antiferromagnetic forces in the compound. At room-temperature viscosity, [C_2_C_1_im]_2_[Co(SCN)_4_] demonstrated low viscosity (145 mPa s^–1^), high ionic conductivity (0.40 S cm^–1^), surface tension (55.37 mN m^–1^), and enthalpy of vaporization (150.4 kJ mol^–1^). The low viscosity and heat of vaporization suggest weak intermolecular interactions between ion pairs.^94^

More recently, Cabeza et al. conveyed a series of metal-containing ILs with chemical structure [C_4_mim]_*x*_[M(SCN)_*y*_], including metals M such as Cr(III), Mn(II), Fe(III), Ni(II), and Co(II).^18,95^ The thiocyanatometallate anions exhibited octahedral coordination, save for [Co(SCN)_4_]^2–^, which had a tetrahedral coordination. The properties of these ILs, such as color, refractive index, thermal behavior, and volumetric properties, depended significantly on the metal–ligand combination. So, for example, the glass transition temperature decreased with increasing atomic size of the metal in the anion complex. The molar volumes of tetrahedral anionic complexes were smaller and similar compared to the octahedral ones. The MIL [C_4_C_1_im]_4_[Ni(SCN)_6_] was a solid at room temperature and showed a lower μ_eff_ (2.84 μ_B_/molecule). Other paramagnetic compounds exhibited weak ferromagnetic interactions, while three-dimensional magnetic ordering was not observed among the compounds studied, the latter being consistent with other [C_4_C_1_im]^+^-based MILs, such as [C_4_C_1_im][FeCl_4_].^18^ The ionic conductivity of these MILs was lower compared to metal-free [C_4_C_1_im][SCN], with [Co(SCN)_6_]^2–^ having the highest ionic conductivity at 298.15 K. Both ionic and molecular conductivity increased with temperature, and the data could be described by the Vogel–Tammann–Fulcher (VTF) or Litovitz equations rather than the Arrhenius type.^95^

**Figure 2:**
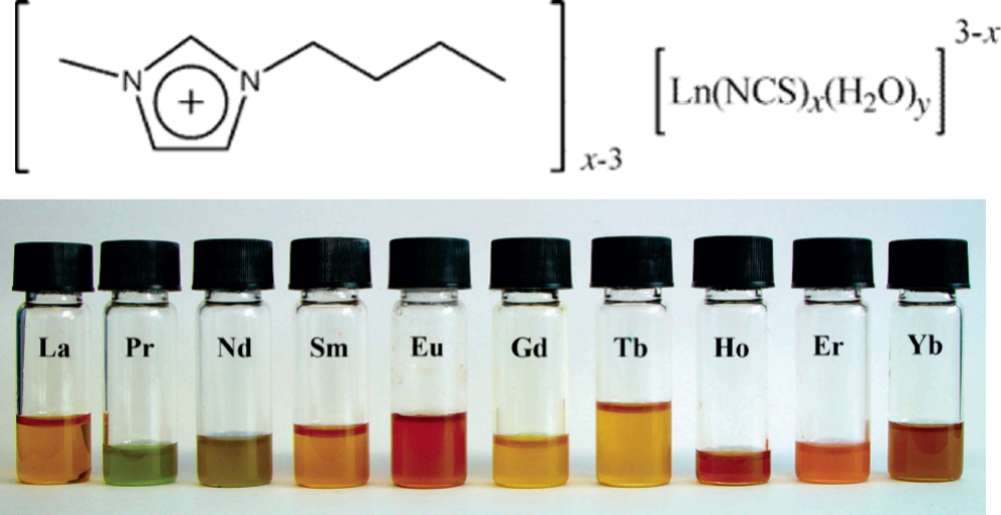
(Top) Structural representation of [C_4_C_1_im]^*x*−3^[Ln(SCN)_*x*_(H_2_O)_*y*_]^3–*x*^ MILs, where *x* ranges from 6 to 8, and *y* from 0 to 2. (Bottom) MIL samples color-coded according to the presence of the lanthanide atoms (Ln = La, Pr, Nd, Sm, Eu, Gd, Tb, Ho, Er and Yb). Adapted with permission from ref (96). Copyright 2006 American Chemical Society.

As for lanthanide-based MILs (see Figure 2), thiocyanatometallate anions [Ln(SCN)_*x*_(H_2_O)_*y*_]^3–*x*^ (where Ln(III) = La, Pr, Nd, Sm, Eu, Gd, Tb, Ho, Er, and Yb, *x* = 6–8 and *y* = 0–2) coupled with the [C_4_C_1_im]^+^ cation exhibited low melting points (below or near room temperature) and high thermal stability (up to 557.15–622.15 K).^96^ The presence of trivalent metal ions in the anion structure of these MILs resulted in the observation of different colors. Strong hydrogen bonding was observed between the isothiocyanate anion and coordinated water molecule, while weaker hydrogen bonds were formed between the C2–H bonds of the imidazolium counterparts and the sulfur atom of the SCN^–^ anions. Normally, high melting points for MILs are related with strong noncovalent interactions, particularly hydrogen bonds, in the ion pair.^97^ However, in this case, the weak H-bond network between ions in the ion pair and the strength of H-bonds involved in water/rare earth thiocyanate complexes may explain the tangible effect in the melting points of [C_4_C_1_im]_*x*−3_[Ln(SCN)_*x*_(H_2_O)_*y*_]^3–*x*^ MILs. These MILs are also soluble in water and polar solvents, such as dichloromethane (except [Ln(SCN)_6_(H_2_O)_2_]^4–^), and miscible with other ILs.^96^ The presence of water in the lanthanide thiocyanate anion structure led to hydrolysis of the latter in the presence of air humidity.^98^

MILs based on metal–thiocyanate complexes exhibit multifunctional characteristics, such as high magnetic susceptibility, long luminescence decay times and high color purity (Figure 2).^4,99^ Additionally, these MILs can exhibit thermochromic properties. For example, the color of [C_2_C_1_im]_2_[Co(SCN)_4_] undergoes reversible changes with temperature. At room temperature, [Co(SCN)_4_]^2–^ has a blue color and tetrahedral coordination, whereas upon cooling to 230 K, its color changes to red and it adopts the octahedral coordination of the [Co(SCN)_6_]^4–^ anion.^100^

#### 2.1.4 Nitratometallates

In the presence of trivalent lanthanide atoms, the nitrate anion acts as a stable ligand with high symmetry, and lanthanides show stronger affinity for O-donor ligands compared to N-donor ligands.^101^ These compounds exhibit intense luminescence, but the presence of water decreases the lifetime due to quenching effects.

A series of lanthanide nitrate complexes ([Ln(NO_3_)_6_]^3–^, where Ln is La(III) or Ce(III), paired with tri- and tetrazolium cations have been synthesized by Tao et al.^101^ Water and several alcohols are soluble in these MILs. The ability of the cation to form a H-bonding network leads to high melting points (around 363 K), although many of them can form MILs with lower melting points (below 298.15 K). The density of these MILs ranges from 1.59 to 2.1 g cm^–3^.^101^

Ji et al. prepared a total of 11 MILs, containing the hexanitratolanthanate anion [La(NO_3_)_6_]^3–^ coordinated with imidazolium cations with long alkyl chains (with 2 to 18 carbon atoms).^98^ Most of these MILs have melting points above room temperature and show high thermal stability. Furthermore, alkyl chains with more than 12 carbon atoms formed ionic liquid crystals.^98^

The preparation and characterization of the hexanitratosamarate anion [Sm(NO_3_)_6_]^3–^ in combination with imidazolium-family cations has been reported.^102^ Compounds such as [C_*n*_mim]_3_[Sm(NO_3_)_6_], where *n* = 4, 6, and 8, were identified as room-temperature ILs due to their low melting points. The lack of symmetry in the cations, their large sizes, and low charge density contribute to weak electrostatic forces between ions, thereby ensuring lower melting temperatures. These ILs exhibit high thermal stability, with glass transition temperatures ranging from 235.15 to 228.15 K. Under UV light, they display orange photoluminescence with longer lifetimes compared to most Sm(III) complexes. The absence of water in the anions and their highly symmetrical structure may prevent quenching.^102^

These MILs, with their intense luminescence, high thermal stability, and desirable melting points, hold promising applications in various fields. However, there are currently no reported studies on their magnetic, physicochemical, and transport properties.

### 2.2 Cation-Based MILs

In cation-based magnetic ionic liquids, the cation structure is doped with a metal atom. Inagaki et al. synthesized various ferrocenium and cobaltocenium ([M(C_5_H_4_R′)(C_5_H_4_R′′)]^+^, M = Fe and Co, R = substituents) and arene-ferrocenium [Fe(C_5_H_4_R′)(C_6_H_5_R′′)]^+^ cations paired with the bis(trifluoromethanesulfonyl)imide ([NTf_2_]^−^) anion.^103^ These compounds mostly exhibited melting points below 298.15 K. The authors found correlations for designing new metallocenium MILs, namely: (i) bulky substituents in ferrocene derivatives lead to higher melting points (>333.15 K) compared to linear ones (<298.15 K); (ii) alkyl chains in cobaltocenium salts have a larger influence on melting points than the anion; and (iii) the asymmetrical backbone of the cation in arene-ferrocenium salts decreases the melting point. Cobaltocenium MILs demonstrated also a remarkable thermal stability (up to 673.15 K). Although ferrocenium-like cations have high molecular weights, in general, their viscosities are less than 50 mPa s^–1^, and viscosity increases with longer alkyl chains. The incorporation of an arene ring in arene-ferrocenium-based cations increases their viscosity, as shown by [Fe-(C_5_H_4_Ethyl)(C_6_H_5_Ethyl)][NTf_2_] with a viscosity of 88.6 mPa s^–1^.^103^ Furthermore, ferrocenium MILs can be easily synthesized through one-step solventless reactions.^104^ Despite their promising properties, ferrocenium-based MILs are unstable in air, while arene-ferrocenium MILs are unstable in light.^103^

Alkyloctamethylferrocenium ([C_*n*_Fc]^+^, where *n* = 2, 3, 5, 5′, 6, 8, 10, and 12) salts with [NTf_2_]^−^, hexafluorophosphate ([PF_6_]^−^), and nitrate ([NO_3_]^−^) anions have also been investigated.^105^ Notice, however, that the salt [C_*n*_Fc][NTf_2_]^−^ is a stable ionic liquid under air but salts with [PF_6_]^−^ and [NO_3_]^−^ anions are not considered ILs due to their melting points above 373.15 K. The melting points of these compounds decrease with increasing alkyl chain length. Crystal structure analysis revealed that ion pairs in salts with short alkyl chains are stacked alternately, while lamellar structures are formed when cations have elongated alkyl chains.^105^

A recent synthesis reported a new MIL based on the nickel cation with chemical structure [Ni(acac)(Me_2_NC_2_H_4_NC_4_H_6_OEtMe][NTf_2_], where acac = acetylacetonate.^106^ This MIL, besides being magneto-responsive, exhibits thermochromic characteristics. The nickel-based cation lowers the melting point of the corresponding MIL below room temperature, with a glass transition temperature of 229 K. The reversible thermochromism of this Ni(II)-based MIL is attributed to the ether side chain. Upon cooling from 353 to 233 K (below the glass transition temperature), the red color slowly changes to orange. Furthermore, the magnetic behavior of the MIL increases with decreasing temperature, suggesting a closed structure for the cationic complex.^106^

**Figure 3:**
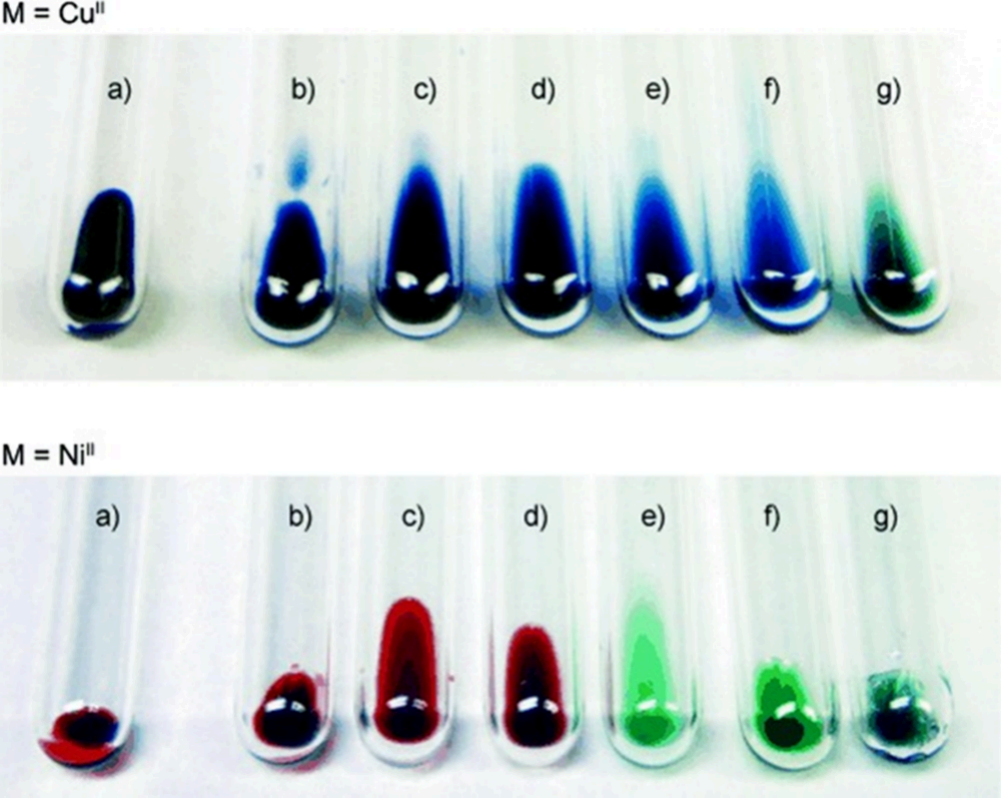
(Top) Cu(II) and (bottom) Ni(II) cation-based MILs (a) before exposure to vapor and following absorption of (b) acetonitrile, (c) acetone, (d) methanol, (e) DMF, (f) DMSO and (g) pyridine. Reproduced with permission from ref (107). Copyright 2012 Wiley.

A reversible color change has also been observed for MILs based on Ni(II) and Cu(II) cation complexes containing diamine and diketonate ligands and the [NTf_2_]^−^ anion (see Figure 3).^107^ The Ni(II)-based cation displays a dark red color, while the Cu(II)-containing MILs are predominantly deep purple in both solid and liquid states. This suggests weak coordination of [NTf_2_]^−^ to the Cu(II) cation moiety. When exposed to organic vapors such as methanol, acetone, dimethyl sulfoxide (DMSO), and dimethylformamide (DMF), the Cu(II)-based MIL gradually changes color within the blue spectrum. In the presence of pyridine vapor, it turns green. Further, DMSO exposure leads to reversible changes in viscosity, with a substantial decrease from 1188 mPa s^–1^ at 298.15 K to 191.3 mPa s^–1^ after DMSO vapor absorption. The color of MIL containing Ni(II) changes from dark red to green upon absorption of organic vapors with high donor abilities like DMF, DMSO, and pyridine. In contrast, in the presence of organic vapors with low donor abilities such as acetonitrile, acetone, and methanol, the color remains red. This color conversion is attributed to spin state changes in Ni complexes, resulting in paramagnetic and diamagnetic states, induced by alterations in their coordination geometry. The reason for this color conversion is that Ni complexes undergo spin state changes, resulting in paramagnetic and diamagnetic states, induced by alterations in their coordination geometry. Consequently, an increase in magnetic susceptibility was also observed after organic vapor absorption.^107^

### 2.3 Dual Paramagnetic-Based MILs

Lately, there have been reports of magnetic ionic liquids containing dual paramagnetic centers functionalized in both the cation and anion structures.^108−110^ These MILs exhibit a significant increase in magnetic susceptibility compared to MILs with single-metal paramagnetic centers.

Wu and Shen,^108^ who have recently developed such a type of MILs, with chemical formula [Ln(TODGA)_3_][Ln(hfac)_4_]_3_, where Ln is Tb, Dy, Ho, Er, Tm and Yn and TODGA is *N*,*N*,*N*′,*N*′-tetra(*n*-octyl)diglycolamide, found precisely a remarkable increase in magnetic susceptibility. These MILs that contain the same lanthanide ion in both the cation and anion result in a 4-fold increase in magnetic behavior compared to MILs with lanthanide halide anions.

Qiao et al. reported a new MIL with dual Co(II)-based paramagnetic centers, [Co(DMBG)_2_][Co(hfac)_3_], where DMBG stands for *N*,*N*-dimethyl biguanide.^109^ This MIL exhibits a higher melting point (341 K) and magnetic susceptibility (7.2 emu K mol^–1^) enhanced over their Co(II) anion-containing counterparts. It also displays a hydrophobic nature and a reddish color. The MIL showed promising analytical performance and can be easily recovered.^109^ Three other recently reported MILs contain Mn in both the cation and anion: [Mn(C_*n*_im)_4_][Mn(hfac)_3_]_2_, where *n* is 2, 6, and 8. [Mn(C_6_im)_4_][Mn(hfac)_3_]_2_ and [Mn(C_8_im)_4_][Mn(hfac)_3_]_2_ are liquid at room temperature, exhibit good hydrophobicity, and can be conveniently applied in microextraction techniques.^110^

To sum up, magnetic ionic liquids with dual-paramagnetic centers demonstrate enhanced magnetism compared to those with single-metal paramagnetic centers. These dual-metal-containing MILs have also shown successful applications in microextraction approaches, such as liquid–liquid microextraction techniques and high-performance liquid chromatography–ultraviolet detection techniques.^109,110^ However, there is still a lack of experimental studies on the physicochemical and structural properties of these compounds.

### 2.4 Metal-Free-Based MILs

Distinguished by their absence of metal elements in the chemical structure, organic paramagnetic-based MILs were first synthesized by Yoshida et al. in 2007.^111^ The paramagnetic properties of these compounds stem from the incorporation of specific anions with organic radical groups, featuring unpaired electrons that contribute to spin. This unique configuration endows them with magnetic responsiveness while being devoid of metal-based components.

The first organic MILs were synthesized using imidazolium cations of varying alkyl chain lengths, namely, [C_2_C_1_im]^+^, [C_4_C_1_im]^+^, [C_6_C_1_im]^+^ and [C_8_C_1_im]^+^ cations, coupled with 2,2,6,6-tetramethyl-1-piperidinyloxyl-4-sulfate (TEMPO-OSO_3_) anions, characterized by a *S* = 1/2 radical spin. Notably, at room temperature, it was observed that the salts containing [C_4_C_1_im]^+^, [C_6_C_1_im]^+^, and [C_8_C_1_im]^+^ cations existed in a liquid state, while the salt featuring the shorter alkyl chain, [C_2_C_1_im]^+^ cation, exhibited a crystalline structure with a melting point at 330.15 K. MILs incorporating TEMPO radical-based anions demonstrated elevated viscosity at room temperature, surpassing 400 mPa s^–1^ at 343.15 K. Their transport properties exhibited an Arrhenius-type temperature dependency, and in the liquid state, a reduction in ionic conductivity was observed with an increase in the alkyl chain length of the cations.^111^

In a more recent development, Nie et al. synthesized MILs based on [TEMPO-OSO_3_] paired with cholinium cations and investigated their physicochemical properties.^112^ The study involved the examination of five alkyl-(2-hydroxyethyl)dimethylammonium ([N_11*n*2_OH]^+^, with *n* corresponding to H or 2, 3, 4, and 5) cations. An observed decrease in density with increasing temperature and elongation of the carbon chain in the cationic component was noted. In aqueous solutions, there was a noted increase in electrical conductivity with rising MIL concentration and temperature, a phenomenon attributed to an augmented number of free charges and reduced viscosity at higher temperatures. Among the MILs studied, the [N_11H2_OH][TEMPO-OSO_3_] compound exhibited the strongest Bronsted acidity, credited to the hydrogen atom bonded to the nitrogen of the cation moiety, significantly enhancing the electron-donating capacity of the compound. Furthermore, a consistent trend was observed across these MILs, where an elongation of the cation’s carbon chain led to an increased acidity. This increase in acidity was explained by the reduced electron-donating ability of the α-carbon with the lengthening of the alkyl chain, resulting in a higher positive charge density on the nitrogen atom and, thereby, facilitating easier proton release from MILs with longer cholinium alkyl chains. Additionally, an increase in magnetic susceptibility correlated with the extension of the cation’s carbon chain was observed. The ability of these MILs to respond to external magnetic fields following the formation of aqueous two-phase systems was also demonstrated. This investigation highlights the significant adaptability of the physicochemical properties of MILs achieved through strategic modification of cationic components, showcasing their potential for tailored applications.^112^

These MIL-based two-phase aqueous systems, incorporating a variety of inorganic salts, also have been investigated for practical applications.^113^ Specifically, the combination of [N_1152_OH][TEMPO-OSO_3_] and potassium phosphate (K_3_PO_4_) has shown high extraction efficiency for alkaloids in natural products. A key advantage of this system lies in its recyclability, demonstrated by the 99.8% recovery of MIL following high-performance liquid chromatography (HPLC) analysis through adsorption methods.

A novel aqueous two-phase system has recently been pioneered using chiral MILs, distinguished by their simultaneous chiral and magnetic properties.^20^ This innovative approach involves a series of amino acid-based MILs, specifically [C_*n*_C_4_im-TEMPO][l-Pro] (where *n* = 2, 3, and 4), a unique combination synthesized by Yao et al. In this study, the MILs were combined with inorganic salts to form an effective aqueous biphasic system tailored for enantiomeric separation. The optimization of extractive resolution conditions proved crucial, resulting in a method that yielded measurable quantities in grams. The major advantage of this system lies in its capability for magnetic and rapid phase separation, a feature that sets it apart from traditional methods reliant on organic solvents. Furthermore, the recyclability of the chiral MILs is noteworthy, maintaining its efficiency for at least six cycles.^20^

Moreover, temperature-sensitive MILs based on polypropylene glycol 1000 [PPG_1000_] have been developed for the enrichment and trace analysis of tetracycline antibiotics in bovine milk.^114^ These metal-free MILs exhibit remarkable changes in their hydrophilic properties with temperature variations, facilitating efficient phase separation and compound recovery. When combined with HPLC, these MILs demonstrated high sensitivity and substantial enrichment factors in a solvent-free, magnetically assisted process. This innovative approach successfully overcame previous challenges associated with phase separation and HPLC analysis interferences, highlighting the efficacy of the methodology. The proposed method not only provides an environmentally friendly solution but also represents a rapid and cost-effective alternative for the detection of trace contaminants in food, showcasing significant potential for practical applications.^8,114^

The metal-free nature of organic MILs broadens their usage, supported by their straightforward synthesis involving few reaction steps, ensuring purity and a single chemical structure. Their recyclability, facilitated by magnetic fields, overcomes the challenge of phase separation seen in traditional aqueous biphasic systems, eliminating the need for centrifugation. Besides, the dual functionality of these MILs, such as temperature sensitivity, allows for simple recovery methods like heating, boosting their practicality and environmental sustainability across various applications.^8,20,114^

## 3 Computational Studies of Magnetic Ionic Liquids: State of the Art and Challenges

The significance of computational approaches in science cannot be underestimated. In the past several decades, remarkable research across diverse fields has become possible due to advancements in computation.^115^ The application of computer calculations becomes mandatory when studying ionic liquids with their virtually unlimited cation–anion combinations, as it is impossible to experimentally explore all the potentially useful ones. Indeed, formulating an appropriate theoretical framework and selecting suitable computational methods can yield meaningful data for the rational design of laboratory experiments. As a result, computer experiments allow for significant resource savings by avoiding costly and time-consuming repetitive laboratory procedures. Additionally, they facilitate a targeted approach toward the most promising candidates for specific applications.

**Figure 4:**
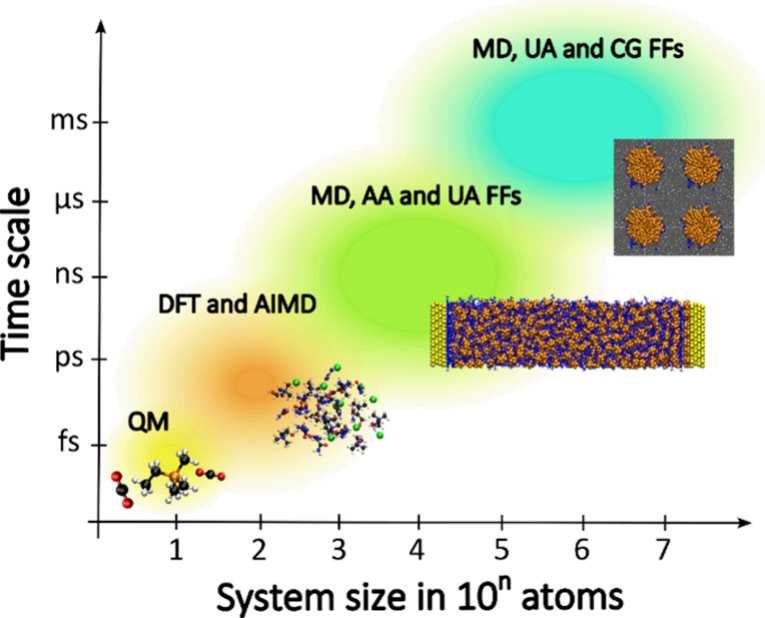
Modeling of IL-based systems: choice of suitable computational methods depending on the sizes of the systems of interest and time scales. Notice that all computational methods depicted can resource to machine learning tools to speed up calculations.

Computer simulations have evolved from being a supplementary analytical tool to interpret experimental observations to a full-fledged separate investigation area capable of providing molecular-level insights into structure–property relationships and designing novel materials from scratch (Figure 4). This transformation has been made possible by the increase in computational power and accessibility of parallel calculations. In addition, a variety of advanced modeling techniques exist today, ranging from theoretical models and first-principle calculations (often called *ab initio* methods) to atomistic and coarse-grained molecular dynamics simulations, including even the incorporation of machine learning tools.^115−119^

At the core of *ab initio* methods lie wave function-based methods like Hartree–Fock (HF) and post-Hartree–Fock methods,^120,121^ as well as density functional theory (DFT) methods.^122^ These methods are commonly referred to as quantum mechanical (QM) methods and have been extensively used to investigate ionic liquids. For example, conformational ordering of [C_4_C_1_im][Cl] has been studied using the post-HF coupled cluster method CCSD(T), revealing multiple stable positions for the Cl^–^ anion around the imidazolium ring, including “in-plane” and “above-ring” positions.^123^ While CCSD(T) along with a large basis set (cc-pVDZ and aug-cc-pVDZ in the referred study) is considered the ultimate standard in *ab initio* methods, it comes with a higher computational cost compared to DFT.^124^ However, the known drawbacks of DFT methods—particularly concerning the description of long-range correlation in hydrogen-bonded liquids, including ILs,^125,126^ can be overcome with commonly applied dispersion correlation schemes,^127^ making them generally preferred. DFT calculations have indeed been successfully employed to assess interionic and ion-molecular association and rationalize vibrational spectra in various ILs and binary mixtures.^128−131^

ILs, with their complex structure and multiple active molecular sites, exhibit various noncovalent interactions such as electrostatic, hydrogen bonding, dispersion, induction, and π–π stacking. Energy decomposition methods, like symmetry-adapted perturbation theory (SAPT), are used to study these interactions and separate them into individual contributions.^132−134^ SAPT, a state of the art supramolecular approach, allows for the breakdown of the systems’ interaction energy into electrostatic, exchange-repulsion, induction, dispersion, and charge-transfer components. This energy decomposition is crucial for predicting and correlating physicochemical properties of ILs, such as conductivity, viscosity or solubility.^135^ As an example, comparative studies using SAPT have highlighted the importance of induction and dispersion interactions in 1,3-dimethylimidazolium chloride, 1-methylpyridinium chloride, and ethyl trimethylammonium chloride ILs, as compared to NaCl.^136,137^ Likewise, a wide range of pyrrolidinium- and imidazolium-based ILs with varying alkyl chains (from methyl to butyl), in combination with eight commonly used anions (Cl^–^, Br^–^, BF_4_^–^, PF_6_^–^, mesylate, tosylate, dicyanamide, and bis(trifluoromethanesulfonyl)imide), were investigated by means of SAPT.^138,139^ The interplay of electrostatic, exchange-repulsion, induction, and dispersion forces was found to govern the interionic distance in the studied ion pairs, as reported in the referenced works. Besides, SAPT calculations are extensively applied in the development of polarizable force fields (FFs) for molecular dynamics (MD) simulations of ILs,^127,138,140,141^ including MILs.^135^

Although helpful in understanding the nature of interactions in ILs, the aforementioned QM calculations are inherently static and currently applicable only to small systems in the gas phase. To observe chemical processes over time, *ab initio* molecular dynamics (AIMD) and classical molecular dynamics (MD) simulations are indispensable.

AIMD computes atomic forces with a DFT approach at every time step, while MD relies on force fields that describe interactions between atomic sites. AIMD is used for systems that are not accessible with QM methods and provides insights into chemical reactivity, vibration properties and hydrogen-bonding dynamics.^142−145^ Classical MD simulations, on the other hand, allow for the study of macroscopic properties of systems consisting of thousands of atoms for extended time periods.^146^

FFs for MD simulations come in different levels of detailing, including all-atom (AA), united-atom (UA), and coarse-grain (CG) approaches. AA models explicitly include all atoms in the simulation, providing a molecular-level representation of the system but with a higher computational cost.^147,148^ UA models redistribute hydrogen atoms’ masses and can be more computationally efficient,^146^ while CG models group multiple atoms into pseudoatoms to tackle larger systems for longer simulation times.^149^ FFs for MD simulations initially used nonpolarizable potentials^147,148,150^ but encountered limitations in predicting transport properties of ILs.^151,152^ To account for polarization effects, polarizable FFs were developed, which include fluctuating induced dipoles, resulting in better predictions of dynamics properties.^117,153−155^

The computational investigation of magnetic ionic liquids (MILs) is still in its early stages due to the lack of FFs and challenges in QM methods related to the large number of electrons in magnetic ions. However, progress is being made with the development of new methods and FFs, paving the way for future studies in this field.

### 3.1 Magnetic Ionic Liquids at Atomistic Level

#### 3.1.1 Interaction Energies

In the study of magnetic ionic liquids, intermolecular forces play a crucial role in predicting and understanding their behavior and providing insights into their nature. While MILs have been experimentally investigated in terms of properties, hydrogen bonding, and Coulomb interactions, there remains a lack of understanding regarding other intermolecular contributions such as halogen–halogen or anion-π interactions.^156^ Obtaining this information through experimental techniques is challenging, making atomistic studies a valuable complement to macroscopic approaches. Hence, quantum mechanical methods help to fill up the puzzles of these phenomena.^127,157^ Likewise, quantifying the interaction energies in MILs allows for a better understanding and prediction of various material properties such as solubility, conductivity, and viscosity. It also facilitates the development of new polarizable force fields capable of accurately capturing the dynamics of MILs. Furthermore, electronic structure calculations offer opportunities to investigate the electronic and magnetic behavior of MILs at a detailed level. By understanding the magnetic properties of MILs, one can get helpful insights into the influence of external factors on their magnetic behavior. This knowledge can then be applied to enhance the prediction and control of the magnetic properties exhibited by MILs.

**Figure 5:**
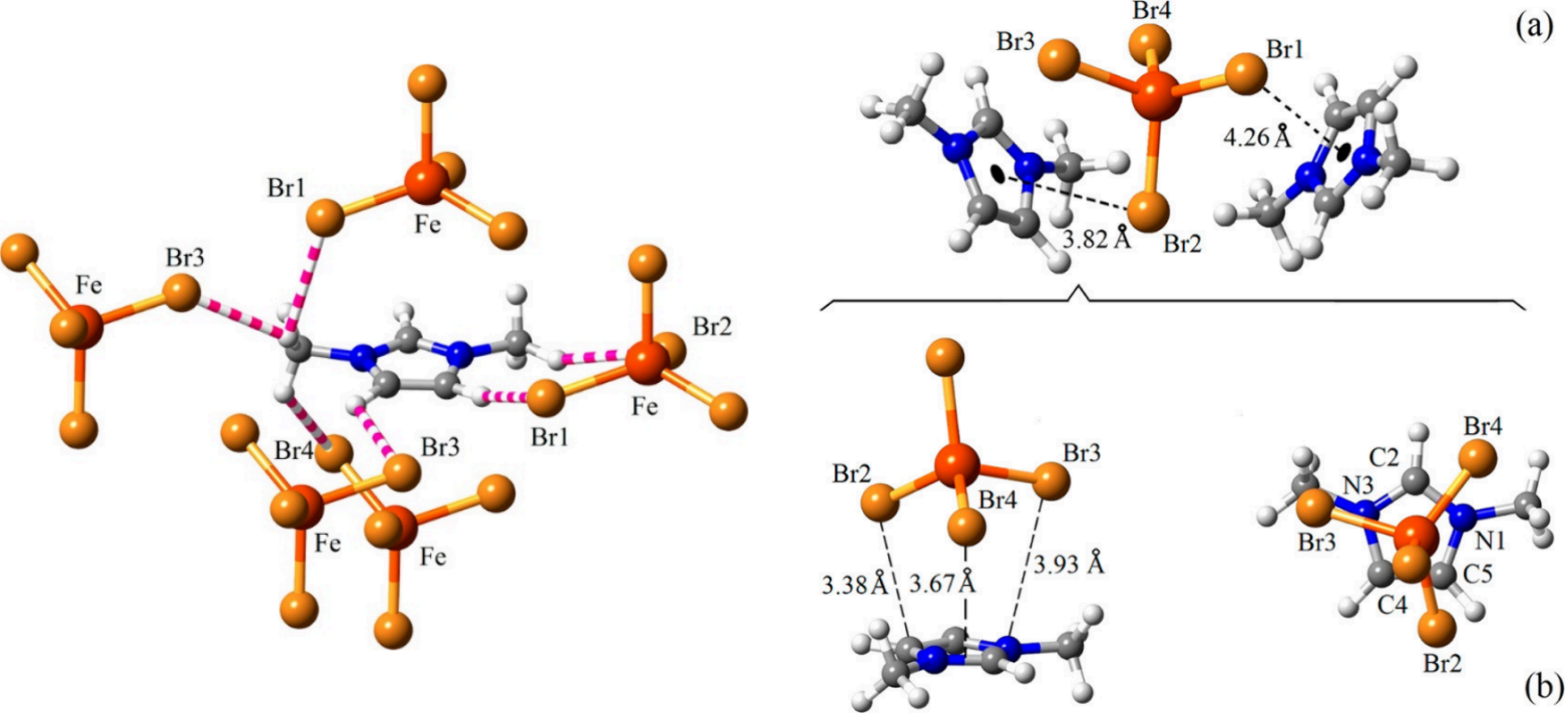
(Left) Illustration of the hydrogen-bond network in the [C_1_C_1_im][FeBr_4_] MIL, denoted by pink and white stripes. (Right) Potential π-*d* interactions between the metal and (a) the central region and (b) the periphery of imidazolium cation. The distances for the strongest interactions are highlighted in Å (angstroms). Reproduced with permission from ref (156). Copyright 2014 American Chemical Society.

For instance, García-Saiz et al.^156^ employed DFT calculations to support the existence of anion-π interactions observed by crystallographic experiments in 1,3-dimethylimidazolium tetrabromoferrate ([C_1_C_1_im][FeBr_4_]). The authors explored two approaches, namely: (i) by computing the “ionization energy” in the gas phase for an isolated ion pair, indicating that anion-π interactions are the most energetically favorable; (ii) by checking their existence in the condensed phase using the projected density of states (PDOS). Through these analyses, they discovered that the wave function can link two [FeBr_4_]^−^ anions across the π orbital above the periphery of the imidazolium ring, as opposed to above the centroid ring (see Figure 5).^156^

Another study by García-Sanchez et al.^158^ resourced to a combination of experimental and DFT methods to investigate the magnetic mechanism of [C_1_C_1_im][FeCl_4_]. By projecting the induced spin density, they confirmed the higher stability of the antiferromagnetic arrangement and observed partial delocalization of spin density on neighboring chloride atoms rather than exclusively on the iron atom. The authors also noted a higher superexchange magnetic interaction between interplanes.

Tian et al.^159^ employed DFT calculations at the B3LYP/LANL2DZ level to investigate the interaction energies of cation–anion pairs and the dipole moments of various *N*-vinyl-3-alkylimidazolium tetrahalogenidoferrate [V_*n*_C_1_im][FeX] (in which *n* stands for butyl, pentyl, hexyl, octyl and decyl, and X stands for Cl_3_Br and Cl_4_) and *N*-vinyl-3-esterimidazolium [Vacim][FeCl_4_] (in which ac stands for ethyl-2-propanoate).^159^ The authors found a relationship between the interaction energies, surface tension, and solubility properties of the various examined MILs. Longer alkyl chains in the [V_*n*_C_1_im]^+^ cation resulted in lower interaction energies and surface tension, while branched alkylacetate chains appended to it led to lower dipole moments and higher interaction energies.

Furthermore, a recent investigation by our group focused on the decomposition of interaction energies in metal-containing MILs—i.e., [C_2_C_1_im][FeCl_4_], [C_4_C_1_im][FeCl_4_], [C_4_C_1_im][FeBr_4_] and [C_4_C_1_im]_2_[SnCl_4_], using SAPT and local energy decomposition (LED) schemes.^135^ The interaction energies calculated using the SAPT0 DFT level, in comparison to the LED DLPNO–CCSD(T) method, are slightly lower by a few kcal mol^–1^ and result in a slight increase in the distance between the centers of mass. However, despite these differences, the LED results follow the same trend as SAPT calculations. Therefore, both methodologies can be used to study the interaction energies in MILs. Electrostatic attractions were found to dominate the cation–anion combinations under study. Interestingly, the variation in the metal atom, reduction of the aliphatic chain, or change of the halide atoms had only a minor effect on the interaction energy of the targeted MILs. However, the presence of two cationic monomers led to a significant increase in their charge and stability.

DFT calculations combined with experimental techniques, like X-ray absorption fine structure and Raman spectroscopy, were employed to examine the atomic-scale structure and temperature effects in MILs, using [C_4_C_1_im][FeCl_4_] as an example. Dissociation reactions of the [FeCl_4_]^−^ anions into bridge-chain [FeCl_5_]^+^ and [FeCl_2_]^+^ structures were observed, indicating an endothermic process.^160^

Moreover, investigations into lanthanide-containing MILs with different cations and anions were carried out using DFT calculations. The heat of formation for these MILs was determined, and the tris(1,5-diamino-4*H*-1,2,3,4-tetrazolium) hexanitratocerate MIL showed promising performance as a propellant.^101^

Balischewski et al. demonstrated the use of DFT calculations to analyze the structures of *N*-butylpyridinium salts composed of single or two metals in the anion structure. DFT calculations were compared to experimental X-ray diffraction data, revealing minimal shifts in lattice planes due to different ionic radii and DFT conditions.^161,162^

Interactions between imidazolium-based ILs and various TEMPO-based radicals have been systematically investigated through DFT calculation at the M06-2X level. Emphasis was placed on the effect of different substituents, including H-bonding (OH) and ionic (N(CH_3_)_3_^+^ and OSO_3_^–^) substituents, on these interactions. The analysis employed, such as natural bond order, energy decomposition, and electron density difference schemes, showed that ionic substitutions in radicals significantly contributed to stronger interactions and subsequently reduced the mobility in ILs. It was found that additional ionic interactions are predominantly electrostatic, influencing the microviscosity and micropolarity of the compounds, as evidenced by electron spin resonance spectra, providing valuable insights for designing task-specific ILs in radical-involved processes.^163^

The COSMO-RS method has been utilized to estimate the properties of MILs.^54^ Imidazolium cations paired with various anions were investigated, and the effect of chain length on interaction energies, dipole moments, and magnetic couplings was examined. It was found that the length of the alkyl chain did not significantly affect the interaction energy of ion pairs, but interaction energies were slightly higher for anions containing chlorine compared to those with bromine. Density and viscosity were also analyzed using the COSMO-RS approach, with calculated values following the experimental trends.^54^

Another study employed a combination of COSMO and DFT methods to predict the isobaric heat capacity (*Cp*) of MILs, and good agreement was found between the calculated and experimental data.^164^ Overall, these studies showcase the valuable applications of DFT calculations and other computational methods in investigating the properties, interactions, and behavior of MILs, complementing experimental approaches and providing deeper insights into these complex systems.

#### 3.1.2 Molecular Dynamics Simulations

Advantages of MD simulations in MILs’ investigation include detailed and accurate information on structure and physicochemical properties, studying systems across broad time and length scales, and predicting performance under different conditions, including the presence of a magnetic field. To the best of our knowledge, the first FF for a MIL was developed in 2015 by Bernardes et al.^165^ The authors proposed and validated an FF for ferrocenium-based MILs, namely, 1-alkyl-2,3,4,5,6,7,8,9-octamethylferrocenium bis(trifluoromethylsulfonyl)imide ([C_*n*_Fc][NTf_2_], where *n* ranges from 3 to 10). Using this model, they accurately reproduced the crystalline structure and enthalpies of fusion for [C_3_Fc][NTf_2_] and [C_4_Fc][NTf_2_] with deviations less than 4.8 kJ mol^–1^. Additionally, the experimental densities of [C_6_Fc][NTf_2_] and [C_10_Fc][NTf_2_] showed good agreement with the simulated results, with deviations less than 1%. Radial distribution function analysis indicated that the strongest atom–atom interactions occurred between: (i) iron atoms of the cation and nitrogen atoms of the [NTf_2_]^−^ anion, (ii) iron atoms of the cations, and (iii) terminal carbon atoms of the alkyl chains. Structural analysis suggested a strong interaction between ferrocenium moieties, in contrast to conventional ILs which show a lack of cation–cation interactions. An interesting finding from this study was that the alkyl side chains in the cations directly interacted with other ferrocenium cores, causing a partial rupture of the polar network and preventing the formation of extended nanosegregated polar–nonpolar domains, commonly observed in other ionic liquids. Interestingly, as the alkyl chain length increased, there were no significant changes observed in cation–anion, cation–cation, and anion–anion interactions. Furthermore, the proposed FF demonstrated transferability with previous parametrizations proposed for ILs.^165^ This enables its combination with other models to study an extensive range of MILs, offering new research opportunities.

In the following years, computer simulations focused only on systems containing tetrachloroferrate anions in the presence of imidazolium cations. For example, Hybrid Reverse Monte Carlo (HRMC) calculations were performed for [C_2_C_1_im][FeCl_4_] and [C_4_C_1_im][FeCl_4_], using a rigid model to represent the molecular structure of the ions.^166^ This method was applied to clarify the unusually continuous structural changes exhibited by [C_4_C_1_im][FeCl_4_] over a wide temperature range (90 to 523 K) without undergoing crystallization. In contrast, [C_2_C_1_im][FeCl_4_] displayed a melting point of 291 K and lacked a glass transition. Specifically, the simulation of the [C_2_C_1_im]^+^ system was carried out at 298.15 K, whereas the [C_4_C_1_im]^+^ system was simulated at 90.15, 298.15, and 523.15 K. The number of ion pairs varied depending on the specific system and temperature. The HRMC results showed that the first coordination shell of the [FeCl_4_]^−^ anion around the [C_4_C_1_im]^+^ cation was more extended compared to that around the [C_2_C_1_im]^+^ cation, leading to the absence of crystallization in [C_4_C_1_im][FeCl_4_]. Additionally, antiferromagnetic interactions between the [FeCl_4_]^−^ ions of [C_4_C_1_im][FeCl_4_] were observed at low temperatures, even in the absence of crystallization.^166^

Daneshvar et al.^167^ also employed MD simulations to study [C_*n*_C_1_im][FeCl_4_], where *n* = 2, 4, and 6. The study aimed to investigate various properties, including volumetric (density and isobaric thermal expansion), dynamic (viscosity, self-diffusion coefficients, and electrical conductivity), and structural properties (radial and spatial distribution functions), at different temperatures ranging from 293.15 to 453.15 K. To carry out the simulations, the authors resorted to a nonpolarizable force field combining OPLS-AA for imidazolium cations and the universal force field (UFF) for [FeCl_4_]^−^ anions. By comparing the computational results with experimental density and viscosity measurements, the performance of the FF was validated. At 293.15 K, the computed density values had relative deviations ranging from 0.7% to 3.8% compared to the experimental values, while the relative deviations for the viscosity values ranged from 15.1% to 22.1%. The authors observed that the different system sizes (729 and 1728 molecules) and cutoff radii (10 and 15 Å) tested did not result in significant differences. The MILs were further assessed for their ionicity using the Walden rule, which establishes a relationship between molar conductivity and fluidity (inverse of viscosity). They found that the ionicity behavior shifted from subionic to superionic at high temperatures, possibly due to the formation of an ideal quasi-lattice. Regarding the structural analysis, the probability of finding an anion around the imidazolium ring turned out to be greater than around the alkyl side chain at 293.15 K. Moreover, this probability increased with the length of the alkyl chain.

Withal, the behavior of tetrachloroferrate-based MILs was simulated in the presence of a 1.5 T external magnetic field to investigate its structural effects.^167^ The results showed that the presence of a magnetic field reduced the intensity of interactions between different atomic sites of the cation and anion in these MILs. Furthermore, the ions were observed to move in opposite directions under the magnetic field, leading to a more homogeneous distribution of the species. According to the authors, these findings hold major implications for the design of viscomagnetic fluids, batteries, and separation processes, in both the presence and absence of a magnetic field. Despite experimental studies indicating an improvement in transport properties (such as viscosity and self-diffusion coefficients) under an applied magnetic field,^75−77,168^ further investigation through MD simulations under the same conditions is still required to better understand the variation of these transport properties.

**Figure 6:**
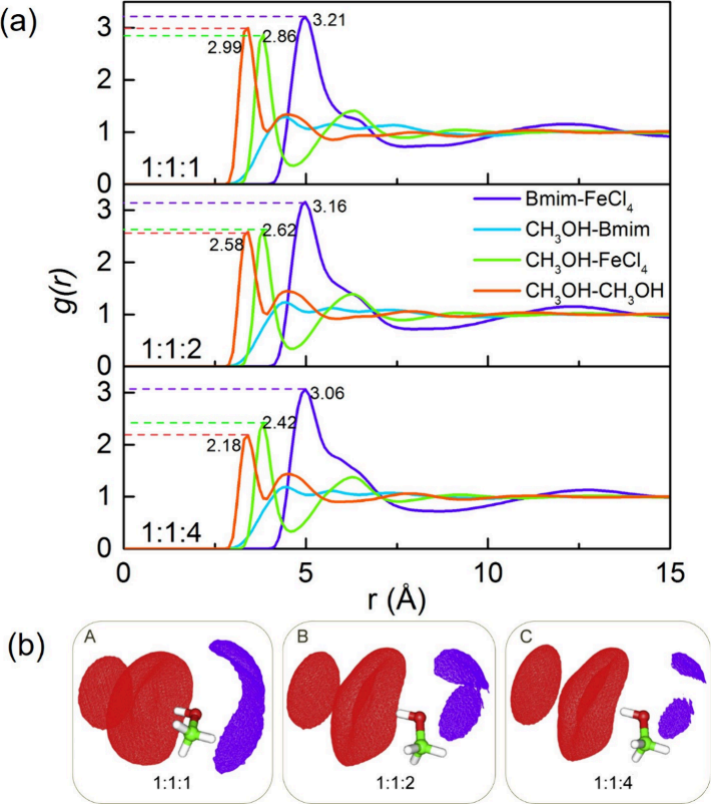
(a) RDFs of center of mass of H-bond network between cation–anion (purple), methanol–cation (blue), methanol–anion (green) and methanol–methanol (red) at different methanol molar ratios (1:1:1, 1:1:2 and 1:1:4). (b) SDFs illustrating the distribution of cation (purple surface) and anion (red surface) species around the methanol molecule at different molar ratios A (1:1:1), B (1:1:2) and C (1:1:4). Reproduced with permission from ref (169). Copyright 2020 Elsevier.

A similar approach was also employed to study [C_4_C_1_im][FeCl_4_] in a binary system containing methanol as a cosolvent.^169^ Radial and spatial distribution functions and the number of H-bonds were analyzed to explain the changes in H-bonding interactions as the molar fraction of methanol varied (1:1, 1:2, 1:4). The radial distribution functions (RDFs) (Figure 6a) indicated that methanol molecules tend to aggregate with each other at lower concentrations, and the relative heights of the methanol–methanol and methanol–[FeCl_4_]^−^ curves decrease as the alcohol concentration rises. Yet, cation–anion interactions remained dominant throughout the dilution process with methanol at higher molar ratios. The number of H-bonds in the MIL–methanol system showed that the C2–H site of the cation (see Figure 1 for reference) is the most favorable binding site for cation–anion interactions. This finding was not common for neat [C_4_C_1_im][FeCl_4_] MIL, as discussed earlier. Furthermore, the H-bonding interaction between the ion pairs is stronger than other interactions. However, as the concentration of methanol increased, the H-bonding interactions between cations and anions weakened, while those between methanol and [C_4_C_1_im]^+^ or [FeCl_4_]^−^ were enhanced, with O–H···Cl H-bonds being the most favorable. Moreover, methanol was found to solvate the [FeCl_4_]^−^ anion more strongly than the [C_4_C_1_im]^+^ cation, likely due to the larger van der Waals radius and lower charge density of the cation. Similarly, the spatial distribution functions (SDFs) (Figure 6b) showed that in the presence of methanol, the distribution regions for [FeCl_4_]^−^ anion (red surface) are larger than those for [C_4_C_1_im]^+^ cation (purple surface).^169^

In 2020, Varela and co-workers^170^ conducted a comprehensive study to understand the interactions of 1-butyl-3-methylimidazolium thiocyanate ([C_4_C_1_im][SCN]) doped with various transition metals including Cr(III), Fe(III), Al(III), Mn(II) and Ni(II), using a combination of MD simulations and DFT calculations. The studied MILs have a stoichiometry of [C_4_C_1_im]_6–*q*_[M^*q*+^(SCN)_6_], where M represents the transition metals that stabilize octahedral complexes with thiocyanate anions. The MD simulations were carried out in the isothermal–isobaric (*NpT*) ensemble (keeping fixed *p* = 1 bar and *T* = 298.15 K) and employing FFs previously reported in the literature. The simulation results for metal-thiocyanate systems indicated higher densities compared to thiocyanate ILs, and these results agreed well with experimental values, with deviations ranging from 3.0% to 3.6% for Cr^3+^-, Fe^3+^-, Al^3+^-thiocyanate systems, and 6.1% for the [Mn^2+^(SCN)_6_]^4–^-containing MIL. Particularly, [C_4_C_1_im]_4_[Ni^2+^(SCN)_6_] was found to be solid at room temperature. Complex stability depended on metal cation charge, with trivalent metals forming more stable complexes than divalent metals. Formation of these octahedral complexes revealed strong nanosegregation in the bulk MILs, segregating polar regions with imidazolium cations and apolar regions with alkyl chains. Furthermore, the absorption spectra calculated for gas-phase complexes matched experimental data and allowed for identification of the most relevant electronic transitions. The authors concluded that imidazolium cations were mere spectators in the electronic transition within the metal–ligand complexes, highlighting their strong nanosegregation behavior.^170^

#### 3.1.3 Phenomena Involving MILs

From an atomistic perspective, understanding the phenomena occurring in magnetic ionic liquids involves examining the interactions between individual ions and molecules in a complex system. Lewis acidic ILs containing metal halide anions ([AlCl_4_]^−^, [CuCl_2_]^−^, and [FeCl_4_]^−^) have shown promising potential for selective sulfur removal due to their thermal stability and fluidity.^171^ Among them, [FeCl_4_]^−^-based MILs have proven to be particularly effective for desulfurization. Thus, for example, experimental studies on 75 ILs revealed that metal halide ILs, especially those containing [FeCl_4_]^−^ with imidazolium cations, were highly efficient in removing sulfur compounds.^172,173^ Moreover, MILs with imidazolium cations and halogenoferrate anions demonstrated high catalytic activity and selectivity for dibenzothiophene extraction (*ca*. 97%). Besides, these [FeCl_4_]-based MILs exhibited also a strong magnetic response, enabling their recyclability for 7 cycles without compromising desulfurization efficiency.^174^ In addition to experimental studies, DFT calculations have also been employed to gain deeper insights into the extractive desulfurization mechanism of organic compounds by MILs.

In their DFT-level study, Ko et al.^171^ have examined interactions between dibenzothiophenes (DBT) and different forms of Fe-containing chloride anions, namely, [FeCl_4_]^−^, [Fe_2_Cl_7_]^−^, and FeCl_3_. The calculations were performed by applying the B3LYP density functional and the 6-31G(d) basis set for C, H, and N atoms, along with the LANL2DZ basis set for Fe and Cl atoms. The study revealed a substantial interaction between DBT and [FeCl_4_]^−^ anion, with an interaction enthalpy (Δ*H*) of −4.5 kcal mol^–1^. An interaction between [Fe_2_Cl_7_]^−^ anion and DBT was also observed, but with a smaller Δ*H* (= −2.4 kcal mol^–1^). Interestingly, the computational results did not support the expected trend that the Fe species with higher nuclearity ([Fe_2_Cl_7_]^−^) would perform better in removing DBT. The authors proposed that this discrepancy might be attributed to the decomposition of [Fe_2_Cl_7_]^−^ into [FeCl_4_]^−^ and FeCl_3_ upon interaction with DBT. Additionally, they suggested that the difference in extraction ability between pure FeCl_3_ and [C_4_C_1_im][Fe_2_Cl_7_] could be explained by the fact that FeCl_3_ formed from [C_4_C_1_im][Fe_2_Cl_7_] is in a solution state, while pure FeCl_3_ exists as a solid.^171^

Martínez-Magadán et al.^173^ utilized DFT-based methods to study the mechanism between [C_4_C_1_im]^+^ and 1,3-di-*N*-butylimidazolium ([C_4_C_4_im]^+^) cations, FeCl_3_, [FeCl_4_]^−^ and [Fe_2_Cl_7_]^−^ moieties, and ethanethiol (the main sulfur-containing compound in gasoline). Molecular reactivity was analyzed by calculating the energy gap between the highest occupied molecular orbital (HOMO) and lowest unoccupied molecular orbital (LUMO) levels. Among the species studied, [Fe_2_Cl_7_]^−^ and FeCl_3_ exhibited the lowest energy gap values, making them the most reactive species. The reactivity was further assessed by calculating the LUMO and HOMO energy differences between pairs of molecules, since that provides an estimate of their thermochemical electronic hopping energy. Ethanethiol reacts favorably with FeCl_3_ due to the smaller energy difference (*E*_LUMO_(FeCl_3_) – *E*_HOMO_(ethanethiol) = −0.938 eV). Moreover, the results show that the excellent performance of tetrachloroferrate anions is favored when there is an excess of FeCl_3_ salt in [C_4_C_1_im][FeCl_4_], as the mixture contained [Fe_2_Cl_7_]^−^ anion (Fe–Cl–Fe bonds are longer and weaker than Fe–Cl bonds) in addition to [FeCl_4_]^−^ anion. The favorable interaction between ethanethiol and [Fe_2_Cl_7_]^−^ anion can be explained by a Dewar–Chatt–Duncanson-like mechanism, i.e., ethanethiol donates electrons to the iron atom of [Fe_2_Cl_7_]^−^ via sulfur, and the iron atom then back-donates electrons to the ethanethiol bond. Nevertheless, in the absence of iron salts, the extractive process occurs via ethanethiol physisorption by cations. This physisorption increases with the size of the *N*-alkyl substituents in cations, mainly due to the influence of van der Waals forces between the substituents of cations and the alkene moiety of ethanethiol.^173^

As later demonstrated by Li et al.,^175^ the role of iron in extractive mechanisms for [C_4_C_1_im][FeCl_4_] MIL and selectivity processes with aromatic sulfur compounds (thiophene, benzothiophene, dibenzothiophene, and alkyl derivatives) are not determined by donation and back-donation mechanisms but rather by charge-transfer effects. Through natural bond orbital (NBO) analysis, the authors found that the coordination number of the iron atom is nearly saturated, making retrodonation unlikely. Instead, the extractive performance of compounds should be influenced by their interaction energies. Regarding [C_4_C_1_im][FeCl_4_]···X (X = aromatic sulfur compounds), they found that thiophene compounds and their derivatives had the lowest interactions with [C_4_C_1_im][FeCl_4_] (ranging from −7.26 to −8.02 kcal mol^–1^), while dibenzothiophene showed the highest interaction (∼10.30 kcal mol^–1^). Hence, the extractive selectivity of [FeCl_4_]^−^-based MILs follows the order thiophene < dibenzothiophene ≈ benzothiophene, and steric hindrance effects should be taken into account for alkyl derivatives. Furthermore, the B3LYP density functional, used in earlier studies, may not adequately capture the attractive forces in ILs systems due to the lack of explicit dispersion corrections.^175,176^ To address this, in this study, the authors have employed the M06-2X density functional along with a diffuse basis set 6-31++G** and an effective core potential described by LANL2DZ for Fe(III) atoms. Based on empirical evidence of magnetic susceptibilities,^177^ a low-spin S = 1/2 state was considered for iron atoms.^175^

DFT calculations have also been used to investigate the reaction mechanism and catalytic activity of various metal ionic liquids ([C_*n*_C_1_im][MCl_3_], where *n* = 2, 3, 4 and M = Cr(III), Fe(III), Mo(III), and W(III)) for the conversion of glucose and xylose to 5-hydroxymethylfurfural (HMF). The transition metal elements were described using the LANL2DZ basis set, while for the rest of the elements the 6-31G+(d,p) basis set was employed, both with the B3LYP hybrid functional. The trivalent iron atoms were considered in a low-spin state, while chromium, molybdenum, and tungsten atoms were considered in a high-spin state. The rate-limiting step in the reaction was found to be the removal of the first water molecule, and the release of the second and third water molecules could be disregarded. According to the changes in Gibbs energy (Δ*G*) determined at 293.15 K in the gas phase, for the [C_3_C_1_im]^+^ cation, the catalytic activity decreased in the following order: WCl_3_ > CrCl_3_ > MoCl_3_ > FeCl_3_ for glucose, and WCl_3_ > MoCl_3_ > CrCl_3_ > FeCl_3_ for xylose. This means that [C_3_C_1_im][WCl_3_] showed the highest catalytic activity in converting both glucose and xylose into HMF among the investigated metal ionic liquids.^177^

**Scheme 1:**
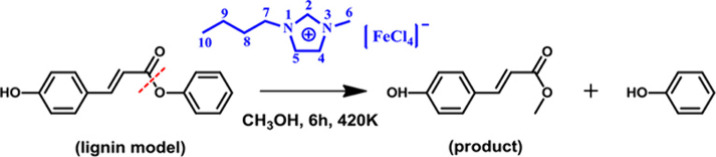
Conversion of Phenyl *p*-Hydroxycinnamate (Lignin Model) to Methyl *p*-Hydroxycinnamate (Product), Using [C_4_C_1_im][FeCl_4_] As Catalyst (Blue Chemical Structure) (Reproduced with permission from ref (178); Copyright 2019 Frontiers)

In a recent study, the lignin model compound phenyl *p*-hydroxycinnamate (PCC) was used to find out the potential of [C_4_C_1_im][FeCl_4_] as a catalyst for depolymerization.^178^ The calculations were carried out at the B3LYP-D3 level, including the initial optimization of the species’ structures, and the Fe(III) atom was described by the SDD basis set, while all other atoms were represented by the 6-31+G(d,p) basis set. The energy of all structures was subsequently recalculated using the M06-D3/6-311++G(d,p) level of theory, save for the iron atom, which maintained the SDD basis set. Based on these calculations, the authors established free energy profiles for potential reaction pathways and determined the most probable pathway for the reaction depicted in Scheme 1.

**Figure 7:**
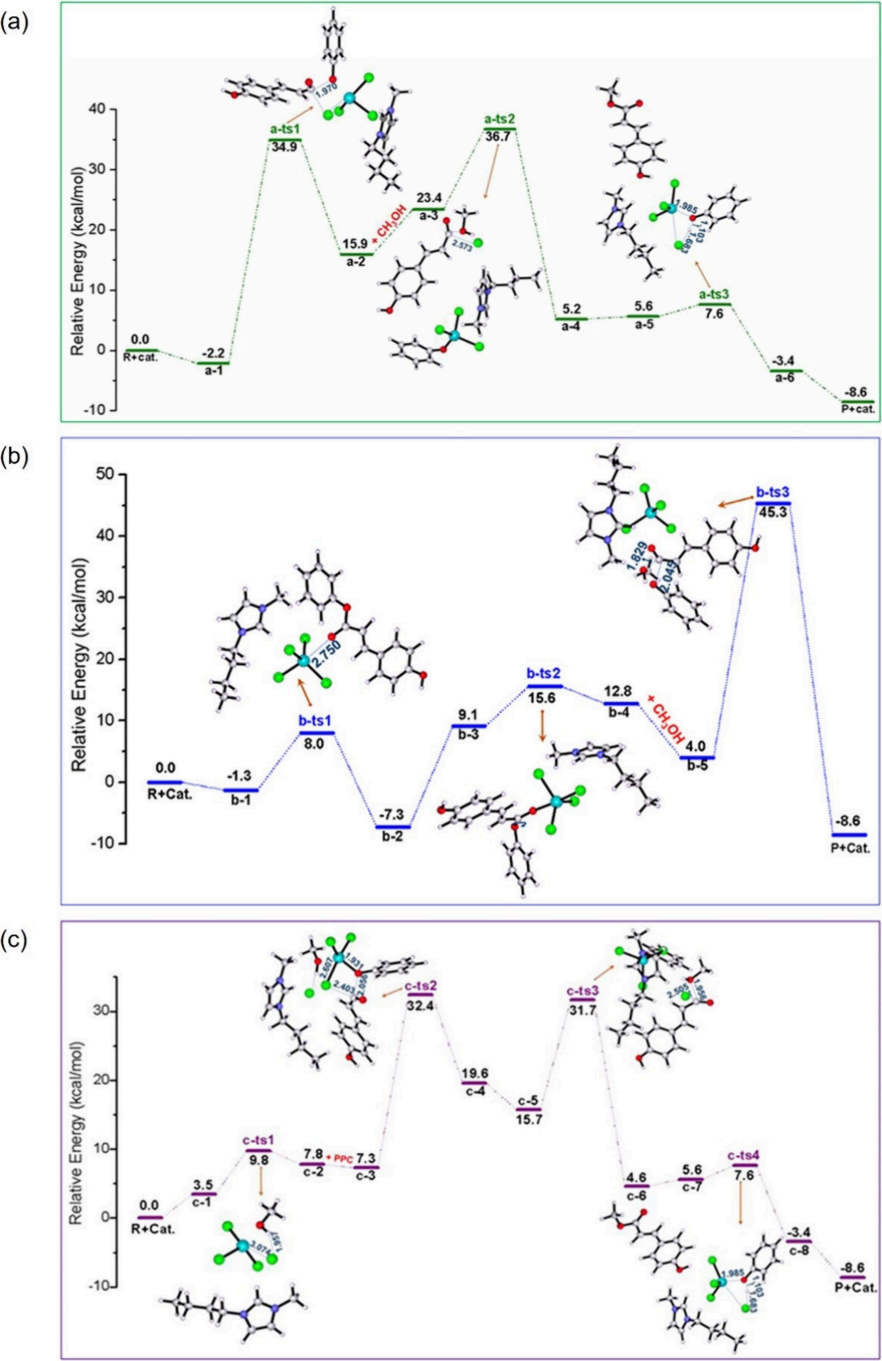
Calculated Gibbs free energy profile with optimized transition state geometries corresponding to the pathways: (a) acyl chlorination process, (b) transesterification processes, and (c) Lewis acid-catalyzed conversion. Distances are in Å. Reproduced with permission from ref (178). Copyright 2019 Frontiers.

Among the three proposed reaction pathways (Figure 7), the Lewis acid catalyzed conversion pathway (Figure 7c) was found to be the most likely to occur. This pathway involves the activation of phenyl *p*-hydroxycinnamate or methanol (acting as both solvent and reactant) by the [FeCl_4_]^−^ anion of the catalyst. Such activation takes advantage of both acyl chlorination and Fries-like rearrangement, leading to lower energy barriers in the initial and rate-determining steps of the reaction. The combination of these two processes contributes to the overall efficiency of the Lewis acid catalytic conversion pathway for breaking down *p*-hydroxycinnamate into methyl *p*-hydroxycinnamate. These findings suggest that the [C_4_C_1_im][FeCl_4_] catalyst shows promise for lignin valorization and provide insights for future studies on the efficient transformation of biomass.^178^ The study thus offers a potential route for the development of effective catalytic processes for biomass conversion and utilization in sustainable applications.

**Figure 8:**
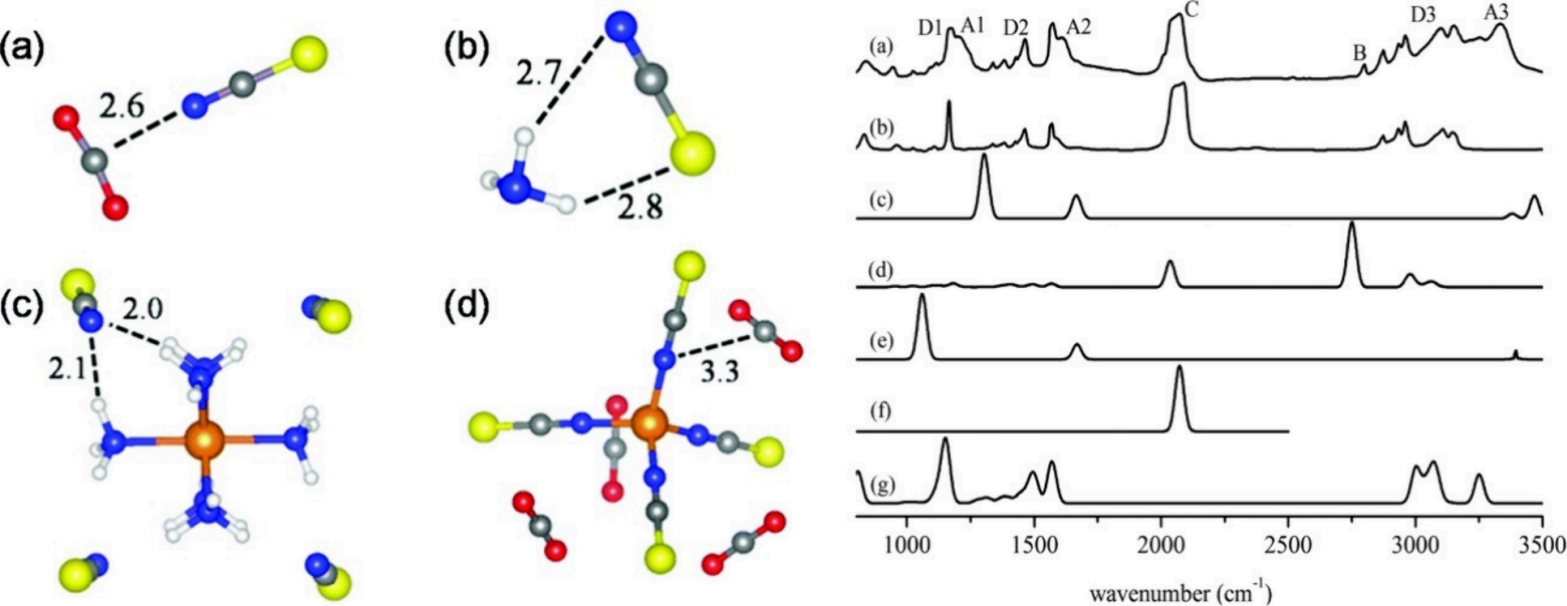
(Left) Mol ecular structures of (a) [SCN]^−^ anion with CO_2_, (b) [SCN]^−^ anion with NH_3_, (c) [Co(NH_3_)_6_(SCN)_4_]^2–^, and (d) [Co(SCN)_4_(CO_2_)_4_]^2–^ anions. Distances between moieties are shown in Å. S, N, O, C, and H atoms are represented by yellow, blue, red, black and white spheres, respectively. (Right) Experimental infrared spectra of [C_4_C_1_im][Co(SCN)_4_] MIL (a) after and (b) before absorption of NH_3_. Computed infrared spectra of (c) [Co(NH_3_)_6_]^2+^ complex, (d) [C_4_C_1_im][SCN] IL, (e) NH_3_, (f) [SCN]^−^ anion, and (g) [C_4_C_1_im]^+^ cation. Adapted with permission from ref (179). Copyright 2018 Royal Society of Chemistry.

Cobalt-thiocyanate anion-based MILs, specifically [C_*n*_C_1_im]_2_[Co(SCN)_4_] (where *n* = 2, 4 and 6), have demonstrated efficient NH_3_ separation, high NH_3_/CO_2_ selectivity, and excellent recyclability.^179^ In comparison to other ILs, besides its reusability, this MIL exhibited significantly higher NH_3_-related activity (e.g., 30 times that of [C_*n*_C_1_im][SCN]). To study NH_3_ absorption and desorption mechanisms, Zeng et al.^179^ performed DFT calculations. The authors optimized the molecular structures of [SCN]^−^ anion, [Co(SCN)_4_]^2–^, NH_3_, and CO_2_ at the TPSS-D3(BJ)/def2-TZVP theory level. PBE0-D3/def2-TZVPP level single-point calculations were then conducted to convey electronic energies. In addition, the gas-phase optimized structures were employed to perform solvation Gibbs energy calculations using the COSMO-RS theoretical approach. The results revealed that, in the presence of metal, NH_3_ undergoes two steps to form a coordinated cobalt complex: (i) NH_3_ molecules replace four [SCN]^−^ anions in [Co(SCN)_4_]^2–^ to coordinate the cobalt center, forming the [Co(NH_3_)_6_]^2+^ complex; and (ii) NH_3_ molecules establish hydrogen bonds with four [SCN]^−^ moieties to further form the following coordinated cobalt complex [Co(NH_3_)_6_(SCN)_4_]^2+^ (Figure 8, left). Each Co–N pair has an estimated distance of about 1.0 Å. The computed Δ*G* of MIL-NH_3_ (−0.6 kcal mol^–1^) indicates that NH_3_ desorption is achievable at higher temperatures. On the other hand, in the MIL-CO_2_ system, CO_2_ and [Co(SCN)_4_]^2–^ do not form chemical bonds, as the shortest distance between them reaches roughly 3.3 Å. This suggests that the interaction between CO_2_ and the cobalt-thiocyanate anion-based MILs is weaker, and thus, CO_2_ absorption is not as favorable as NH_3_ absorption.

In order to investigate the mechanisms responsible for NH_3_ absorption by [C_4_C_1_im]_2_[Co(SCN)_4_], the authors compared computed infrared spectra with experimental ones (Figure 8, right). After NH_3_ absorption, four new bands (A1, A2, A3, and B) can be observed in the experimental IR spectrum of [C_4_C_1_im]_2_[Co(SCN)_4_] (Figure 8(a), right). The peaks A1 to A3 correspond to the vibrational modes of NH_3_. Additionally, Figure 8(c) and (f) show the IR spectra of [Co(NH_3_)_6_]^2+^ and free NH_3_, respectively, which further confirm the chemical absorption of NH_3_. It also can be seen that peak B corresponds to the C–H stretching mode of the imidazolium ring (Figure 8(d), right), while peak C corresponds to the C–N stretching mode of the anion (Figure 8(f), right). Peaks D1 to D3 correspond to the stretching node of the C–H groups in the CH_3_- and CH_2_- groups, as well as the C–H of the cation. Based on these results, the authors concluded that NH_3_ replaces the thiocyanate in the [Co(SCN)_4_]^2–^ complex through H-bonds upon absorption. As a result, the N atom of [SCN]^−^ interacts with the C–H group of the imidazolium cation via the H-bond network. These acid–base interactions between the metal center-ligands and NH_3_ capacity and recyclability are achieved by the cobalt thiocyanate-based MIL.^179^ This insight thus provides valuable information for the design and optimization of metal ionic liquids for efficient NH_3_ separation and utilization in various applications.

Recently, Goloviznina and Salanne have investigated the electrochemical and catalytic properties of TEMPO and its oxidized variant, TEMPO^+^, within diverse ILs.^180^ In these TEMPO/ILs systems, it was discovered that the two forms exhibit distinct solvation environments in ILs, with TEMPO forming hydrogen bonds with cations of ILs and TEMPO^+^ demonstrating a tendency to form weak hydrogen bonds with the anion moiety. ILs characterized by smaller cations and hydrophobic anions with low basicity tend to accelerate the oxidation rates of TEMPO. Conversely, the reduction process of TEMPO^+^ proved to be more efficient in the presence of larger, less acidic cationic components. This observation underscores the role of solute–solvent interactions in the stabilization of both TEMPO and its oxidized counterpart within IL environments. Notably, ILs with lower viscosity are identified as particularly advantageous for these processes, promoting more effective mass and electron transfer.^180^

### 3.2 Future Perspectives in Computational Studies of MILs

Despite the progress made in the development of task-specific ionic liquids, including MILs, there is still a lack of detailed information about their properties and structures. Experimental studies since 2004 have aimed to characterize these compounds, but thermodynamic properties (e.g., heat of vaporization) and transport properties (e.g., viscosity, ionic conductivity, and self-diffusion coefficients) are still inadequately explored, hampering the validation of computational models for MILs.

Recently, computational studies have focused on electronic structure calculations to explore the properties of MILs. However, the open-shell nature of these systems, caused by the presence of paramagnetic atoms, poses additional challenges. Modeling the electronic structure and magnetic properties of MILs, particularly those with a high number of electrons like rare earth metals, is difficult. To address this problem, the primary objective in studying open-shell systems is to employ a basis set that can accurately describe their electron density.

Regarding SAPT, where the electronic wave function is described by a single determinant, only calculations at the zeroth-order in intermolecular correlation can handle closed- and open-shell monomers. The highest-order SAPT calculations are still restricted to interactions between monomers with closed electron shells.^181^ However, in the case of paramagnetic atoms with unpaired electrons, multiple electronic configurations are possible, leading to more complex electronic wave functions in open-shell systems like MILs. Choosing an appropriate reference state becomes a significant challenge in such systems since there is no clear choice like in closed-shell systems where the reference state is usually the corresponding Hartree–Fock wave function, represented as a single determinant. Consequently, different reference state choices can yield different outcomes, making it difficult to determine the most suitable one. Another problem is the treatment of electron correlation in open-shell systems. While the SAPT high-level method incorporates higher-order contributions to the interaction energy, such as exchange and dispersion, it is only available for closed-shell systems, which further complicates the accurate treatment of electron correlation in MILs.

Future research on MILs will need to overcome these challenges and strive to meet more demanding benchmarks. As seen in a recent study, the popular dispersion-corrected B3LYP-D3/D4 schemes applied to MILs may not be as accurate for open-shell systems as the Minnesota functional, M06-2X, or other exchange-correlation functionals like ωB97M-V, and ωB97M-D3(BJ).^182^ Moreover, comparisons with closed-shell ILs using SAPT0 to high orders are essential for checking the interaction decompositions in MILs. For example, in our recent study, SAPT2+ does not produce any change in the trend of interactions for the [C_4_C_1_im][ZnCl_3_].^135^ Yet, comparing the total energy obtained at the SAPT0 level with the energy from the CCSD(T) method can further validate the computational results.

Meanwhile MD simulations have played a vital role in advancing the understanding of ionic liquids and their mixtures, from both physicochemical and structural perspectives, as well as their practical applications. However, there is still limited research on MILs. One of the reasons for this scarcity is the lack of well-developed force fields for cation- and anion-based MILs. To address this, new force fields need to be parametrized and validated for a broader range of cation and anion classes, especially for anion-based families, considering that several transferable FFs are already available for the most common cation species (see Figure 1).^148,150,151,154,183^ Furthermore, UFF parameters^184^ have been successfully applied to the iron atom,^167^ and nonbonded parameters of Lennard-Jones types are available for all elements of the periodic table. This was considered when developing the OBGMX tool,^185^ which generates topologies for MD simulations compatible with the GROMACS software package.

Additionally, one significant challenge in simulating ILs, including MILs, is their sluggish dynamics. One approach that has been used to improve dynamics of ILs is the use of polarizable force fields.^117,153,183^ These models explicitly represent the electronic degrees of freedom of atoms in the system by using induced dipoles connected by a spring-like potential. Although polarizable FFs require extensive parametrization using first-principles calculations, new strategies have been developed to modify additive force fields by evaluating individual energy components, such as induction and dispersion, and scaling the Lennard-Jones terms.^140,153,183^ This approach allows for the establishment of polarizable FFs without the need for expensive calculations, proving advantageous for simulating ionic liquids, including MILs, in the future. In addition, to address the slow dynamics of ILs and MILs, accelerated dynamics methods like hybrid Monte Carlo simulations or nonequilibrium molecular dynamics can be employed. These techniques modify equations of motion to enable faster relaxation times, leading to more efficient simulations,^186,187^ and have already been successfully applied in other fields.^188^

Furthermore, it is plausible to assume that an external magnetic field can influence the transport properties of MILs. Experimental studies have indeed shown that the viscosity and mobility of MILs can be enhanced in the presence of such fields.^74,75,77^ However, the underlying reasons for this effect are not yet fully understood. Molecular dynamics simulations provide a valuable tool to investigate and quantify these effects, including the structural changes in MILs induced by the presence of a magnetic field (e.g., through radial distribution functions). Moreover, molecular dynamics software packages such as NAMD^189^ and LAMMPS^190^ already implement an external magnetic field, thus facilitating the study of MILs in such conditions.

## 4 Summary and Outlook

This Review summarizes the main “families” of magnetic ionic liquids, highlighting their physicochemical, magnetic, chromic, and luminescent properties, with a particular focus on potential applications, such as extraction and separation processes.

MILs exhibit a range of properties and applications, primarily attributed to the incorporation of paramagnetic species from transition metals, rare earth elements, or organic radicals. These species impart MILs with high magnetic moments, positive magnetic susceptibilities, luminescence, and stimulus-responsive behavior.

Extensive experimental research showcases the broad diversity and tunable properties of MILs. Anion-based MILs, for instance, display distinct thermal and magnetic properties, along with chromic behavior and luminescence, while cation-based MILs feature unique attributes, notably lower viscosity. Noteworthy advancements include dual-paramagnetic-based MILs, which enhance magnetic susceptibility, and metal-free MILs employing organic radicals, thereby expanding research possibilities into innovative systems like MIL-based two-phase aqueous systems, with remarkable versatility, for example, in extraction processes. Future experimental studies should explore MILs in binary mixtures, aiming to achieve viscosity reduction, thereby enhancing MILs’ dynamics and expanding their applications.

The Review also specifically addresses major advancements in computational approaches for studying MILs. Particularly, DFT calculations have proven essential in understanding the interactions among ions in MILs, contributing to the knowledge of intermolecular forces, electronic and magnetic properties, and chemical reaction mechanisms. In contrast, MD simulations have provided valuable insights into the structure and physicochemical properties of MILs, enabling the prediction of their properties under different conditions across a wide range of time scales. These studies offer detailed insights into spin density delocalization, complex formation, and catalytic activity of the studied MILs. The analysis of atomic structure and the effects of temperature on MILs are additional crucial aspects addressed through MD simulations, offering detailed information about ion-pair interactions, structural organization, volumetric and dynamic properties, as well as the influence of solvents on MILs.

Notwithstanding, it is important to acknowledge that research on MILs from a computational standpoint is still in its early stages. The following are only a few critical aspects that must be considered on future computational studies of MILs: first, it is essential to address the challenges posed by the open-shell nature of MILs, specifically, developing and refining computational methods in order to accurately describe the electronic structure and magnetic properties of MILs, especially those containing rare earth metals. This includes the selection of appropriate basis sets and reference states, as well as the treatment of electron correlation in open-shell systems. Second, the development or refinement of force fields for MD simulations of MILs is required. These advancements will enable more accurate and efficient simulations of MILs’ properties, facilitating a better understanding of their behavior. Furthermore, the continuous increase in computational power foresees a growing application of polarizable force fields in MD simulations and ab initio MD for a more in-depth study of MILs. Third, incorporating the effects of external magnetic fields into MD simulations will provide valuable insights into the transport properties of MILs and their structural changes.

The dynamic interplay, arising from experimental observations and computational modeling, is deemed essential for validating computational models and refining theoretical frameworks. Ultimately, the integration of experimental findings with sophisticated computational techniques is anticipated to play a pivotal role in unraveling the complexities of MILs and harnessing their full potential.
